# Encoding neural representations of time-continuous stimulus-response transformations in the human brain with advanced deep neural networks

**DOI:** 10.1162/IMAG.a.1142

**Published:** 2026-03-16

**Authors:** Sabine Haberland, Hannes Ruge, Holger Frimmel

**Affiliations:** Institut of General Psychology, TUD Dresden University of Technology, Dresden, Germany

**Keywords:** deep neural networks, encoding models, arcade games, neuroimaging, LSTM and fMRI

## Abstract

Human behavior arises from the continuous transformation of sensory input into goal-directed actions. While existing analytical methods often break time into discrete events, the stages and underlying representations involved in stimulus-response (S-R) transformations within time-continuous, complex environments remain incompletely understood. Encoding models, combined with deep neural networks (DNNs) for feature generation, offer a promising framework for capturing these neural processes. While DNNs continue to improve in performance, it remains unclear whether these advances translate into closer alignment with human cognitive mechanisms. To address this, we collected fMRI data from participants (N = 23) as they played arcad video games and used DNN-based encoding models to predict human brain activity. We compared the prediction accuracy of features from three DNNs at different stages of development within our encoding model. The results show that the most advanced DNN provides the most predictive feature space for neural responses, while also revealing a closer hierarchical alignment between its internal representations and the brain’s functional organization. These results enable a more fine-grained characterization of time-continuous S-R transformations in high-dimensional visuomotor tasks, progressing along the dorsal visual stream and extending into motor-related regions. This approach highlights the potential of machine learning to advance cognitive neuroscience by enhancing the investigation of ecological valid experimental tasks.

## 1 Introduction

The human brain is capable of rapidly processing complex, dynamic information of the environment and responding flexibly with appropriate actions, whether it is catching a flying ball or navigating through traffic. The study of these underlying neural processes has been a central focus of research and a key source of inspiration for the development of artificial neural networks in machine learning. Even though neuroscience and machine learning have advanced rapidly in recent decades, it is only recently that the two fields have begun to exchange concepts and benefit from each other’s progress (Naselaris et al., 2018). These brain-inspired neural networks are increasingly being used as computational tools to better understand neural processes themselves (Cichy & Kaiser, 2019; Kriegeskorte, 2015). While machine learning has primarily focused on improving performance to automate complex tasks, it remains unclear whether these advancements can be translated back into neuroscience, specifically, whether they can contribute to a more fine-grained investigation of stimulus-response (S-R) transformations (Botvinick et al., 2020).

Factorial experimental designs, which are typically constrained by a low-dimensional stimulus space and trial-based structure, have provided valuable insights into the neurofunctional architecture of the human brain. However, they may not fully capture the complexity of real-world processing, which limits their ecological validity. The encoding model approach has emerged as a promising method for modeling neural activity patterns evoked by complex stimuli, like naturalistic images, by using high-dimensional stimulus feature spaces such as hand-crafted features based on a Gabor wavelet pyramid (GWP) (Kay et al., 2008; van Gerven, 2017).

A breakthrough has been achieved by combining encoding models with deep neural networks (DNNs). In this approach, the activations of the DNNs serve as nonlinear representations of stimulus features for the encoding model. Studies have shown that convolutional neural networks (CNNs), which achieve human-level accuracy in object classification, can be used to generate suitable stimulus features. This enables a more precise analysis of voxel activity along the ventral visual stream when subjects are presented with naturalistic images than features derived from GWP models (Güclü & van Gerven, 2015). Additionally, studies have revealed a hierarchical structure in these representations, suggesting that the layered architecture of the DNNs captures the stages of visual processing and complexity observed in the human brain along the ventral visual stream (Cichy et al., 2016; Eickenberg et al., 2017; Güclü & van Gerven, 2015). In these experiments, subjects passively viewed visual stimuli and were not required to perform specific actions. This approach allows the investigation of the ventral visual stream, which extends from the primary visual cortex in the occipital lobe to the temporal lobe. The ventral stream is primarily responsible for generating detailed visual representations, which are important for object recognition and categorization (Goodale, 2014; Grill-Spector & Weiner, 2014). In contrast, the dorsal visual stream serves as a second pathway, extending from the primary visual cortex to the posterior parietal lobe (Goodale & Milner, 1992). Studies have demonstrated that this stream is activated when visual information must be transformed into motor actions (Goodale, 2011). Moreover, recent studies have shown that S-R transformations in visuomotor tasks involve functional connectivity between the posterior parietal cortex (PPC) and the premotor cortex (PMC) (Brovelli et al., 2015; Creem-Regehr, 2009; Kravitz et al., 2011). Along the dorsal stream, studies have also demonstrated a hierarchical similarity between stimulus processing in the brain and representations in DNNs during action recognition (Güclü & van Gerven, 2017a). Due to conceptual limitations, the dorsal visual stream has remained underexplored, and its neural representations are less understood than those in the ventral stream. This is partly because sensorimotor integration involves complex, dynamic functions that are difficult to capture with traditional neuroimaging and often require elaborate behavioral paradigms. Advances in machine learning, however, provide new ways to study these processes.

These advancements, particularly the combination of deep learning with reinforcement learning (RL), have led to the development of deep Q-networks (DQNs). DQNs can solve tasks involving complex, time-continuous stimuli that require motor responses at or above human-levels (Mnih et al., 2015). A DQN approximates a Q-function, which estimates the expected cumulative discounted reward for taking an action in a given state, aiming to maximize the total reward over time. DQNs are not only technically powerful function approximators for complex environments, but are also theoretically grounded in RL, which has a long-standing history as a framework for understanding reward-based learning and decision making in the brain (Niv, 2009; O’Doherty et al., 2015; R. S. Sutton & Barto, 1998). In particular, temporal-difference learning models have been shown to closely align with dopaminergic reward prediction error signals and to account for behavioral adaptation across a wide range of experimental paradigms (O’Doherty et al., 2003; Schultz et al., 1997). Building on these principles, DQNs can be viewed as an extension of traditional RL models, enabling learning and decision making to scale from simplified laboratory tasks to environments characterized by high-dimensional, time-continuous sensory inputs and complex action demands (Botvinick et al., 2020; Mnih et al., 2015). When DQNs are integrated into a DNN-based encoding model, this approach has successfully predicted human behavior during arcade gameplay (Haberland et al., 2025; Mohr et al., 2019). It also provides ways to explore functional similarities and model neural processes underlying S-R transformations in visuomotor tasks, as demonstrated by studies that investigated brain activity in participants playing video games (Cross et al., 2021; Kemtur, 2023; Paugam, 2024). However, this video game paradigm has received limited attention so far. The results of these studies demonstrate that even basic DQNs can capture neural activity underlying human interaction in complex, dynamic environments.

In recent years, the development of DQNs has progressed significantly and enabled them to achieve superhuman performance across various tasks (Espeholt et al., 2019). At the behavioral level, it has already been demonstrated that advancements in machine learning can be leveraged to improve prediction accuracy when modeling human behavior in complex, time-continuous experimental tasks at a fine-grained temporal scale (Haberland et al., 2025). Building on previous findings, we aimed to investigate whether advancements in machine learning can capture neural representations underlying the steps of S-R transformations in complex, time-continuous tasks, thereby enabling a more detailed characterization and localization of these processes.

Therefore, we analyzed fMRI data from N=23 participants, each measured on three separate days while playing the arcade games Breakout, Space Invaders, and Enduro (see Supplementary Fig. S15). The three games represent different gameplay mechanics. We applied a DQN-based encoding model approach to predict the underlying fMRI activation patterns. To assess progress in RL, we compared two recently developed DQNs, Ape-X (Horgan et al., 2018) and SEED (Espeholt et al., 2019), against a baseline DQN (Mnih et al., 2015), as feature-generating mappings within an encoding model, comparing their prediction accuracy for voxel activity (see Table 1 for an overview of the three DQNs). While all three DQNs are capable to play arcade games after sufficient training, with SEED, the most advanced of the three DQNs, clearly showing the highest gaming performance (see Table 2), they differ in their architectures and training methods. The baseline DQN is a standard feed-forward CNN. Although Ape-X and SEED share structural similarities with the baseline DQN, they incorporate additional architectural components and leverage larger amounts of training data through parallel sampling and optimized hardware utilization. Ape-X and SEED integrate a dueling architecture and SEED further introduces a long short-term memory (LSTM), a recurrent neural network (RNN) that incorporates past experiences into decision-making (Hochreiter & Schmidhuber, 1997). These advancements result in a significant increase in performance that surpasses human-level performance in all three games.

**Table 1.** Overview of the three DQN architectures, emphasizing their similarities and differences.

Baseline DQN	Ape-X	SEED
**Input:**		
Received a stack of four consecutive video frames and a terminal state flag as input.	Received a stack of four consecutive video frames and a terminal state flag as input.	Received a stack of four consecutive video frames, a terminal state flag, the achieved reward as input, and maintains past game states via an LSTM.
**Architecture:**		
Vanilla feed-forward architecture: after convolutional layers (layers 1-3), a fully connected layer with 512 units is added (layer 4) before the output layer.	Dueling network architecture (Wang et al., 2016): after convolutional layers (layers 1-3), two separate streams with 512 units each estimated the state value and action advantages (layer 4), which are combined in the output layer.	Recurrent dueling architecture: after convolutional layers (layers 1-3) and a fully connected layer, an LSTM module (Hochreiter & Schmidhuber, 1997) with a single LSTM cell with 512 units is applied to capture temporal context for future decision-making, enabling the model to determine which information is relevant. This is followed by a dueling architecture comprising two separate streams of 512 units each (layer 4) and the output layer.
**Training methods:**		
The gaming experiences were stored in a replay buffer (Lin, 1992), and random batches were sampled to train the network.	The training was distributed across multiple actors and a learner. Actors collected experiences in parallel environments, which were prioritized for replay (Schaul et al., 2016). Double Q-learning (van Hasselt et al., 2016) and multi-step bootstrap targets (R. Sutton, 1988) were applied.	The training was coordinated by a centralized learner responsible for inference, trajectory accumulation, and the computation and updating of model parameters, while actors executed actions in multiple environments and sent their observations back to the learner. It combined Q-learning with policy gradient methods and integrated double Q-learning, multi-step targets, prioritized replay, value function rescaling (Pohlen et al., 2018), and V-trace (Espeholt et al., 2018). Optimized task distribution, efficient resource utilization, and advanced parallelization strategies improved training stability and scalability.
**Training time:**		
Three days of training and approximately 60 million environmental frames; training was stopped once performance no longer improved.	Eight days of training and approximately 800 million frames; training was stopped once performance no longer improved.	Due to limited computational resources, pretrained network checkpoints from (Espeholt et al., 2021) were used, corresponding to approximately 40 billion frames.
**Gaming performance:**		
Achieved human-level performance in Enduro.	Exceeded human performance in all three games.	Exceeded human performance in all three games.
**Code:**		
The implementation follows the theoretical framework presented in (Mnih et al., 2015).	The code is based on the GitHub repository (Neka-nat, 2020), with modifications for integration into our data analysis pipeline and the arcade environment.	Code and checkpoints were obtained from the GitHub repository (Espeholt et al., 2021).

**Table 2.** Comparison of gaming performance between the three DQNs and human players.

	Baseline DQN	Ape-X	SEED	Humans	Human expert (Mnih et al., 2015)
Breakout	43.1	469.8	768.4	69.5	31.8
Space invaders	761.3	2571.1	3626.6	1346.6	1652
Enduro	373.1	500	500	312.5	309.6

DQN performance is reported as the score per episode, averaged across 50 episodes. Human performance is represented by the average game score per episode achieved by the participants in the experiment. The human expert scores are taken from Mnih et al. (2015). In Enduro, the maximum achievable score in our experiments is limited to 500 due to the episode length of 18,900 frames.

Using the DQN-based encoding model, we replicated previous findings showing that this method is well-suited for modeling S-R transformations in time-continuous and complex environments. Building on these results, we confirm our hypothesis that recent advances in machine learning enable a more fine-grained characterization of neural representations, particularly in higher-level cognitive control regions, making this approach even more valuable for future investigations of temporally structured behavior. By comparing the spatial representations in the brain with those in the DQNs, we uncover a hierarchical correspondence between DQN layers and visuomotor processing stages in the dorsal stream during visuomotor tasks. These findings highlight the potential of machine learning to advance neuroscience.

## 2 Methods

### 2.1 Participants

The participants (N=23 , age range: 19−35 , 9 female, 14 male) were right-handed, neurologically healthy, and had normal or corrected-to-normal vision. They were recruited from the population of the TUD Dresden University of Technology. Due to incomplete data collection from one participant, only N=22 datasets were available for the Space Invaders task. None of the participants participated in a similar behavioral experiment from Haberland et al. (2025). Participants’ prior gaming experience was not a criterion in our recruitment process. They were compensated with 10 EUR per hour, along with an additional variable bonus based on their game score (range: 3.87 EUR–15.98 EUR per game).

### 2.2 Experimental design and task

#### 2.2.1 Ethics statement

The experimental protocol was approved by the Ethics Committee of the TUD Dresden University of Technology (EK 359072019) and was conducted in accordance with the Declaration of Helsinki. All participants were informed about the study’s objectives and procedures and provided written informed consent before participation.

#### 2.2.2 Experimental paradigm and procedure

Each participant attended three separate appointments on three different days. On each day, one of the three arcade games, Breakout, Space Invaders, or Enduro, was played (see Supplementary Fig. S15). The games were selected in a pseudo-random order. Each appointment began with the participants reading the gaming instructions for the chosen game, including finger placement, controls, objectives, and scoring. Then, they trained the game for 35 minutes in a mock MRI scanner to become familiar with the environment and the game. After training, participants transitioned to the MRI scanner for the main experiment. Following a T1-weighted high-resolution anatomical scan, a field map, and a localizer, they played each of the three games for five sessions. Each session lasted 7 minutes of gameplay, corresponding to 18,900 frames. Multiple episodes of the game could be played within a single session. An episode refers to the time from the start of the game until ‘game over’. Participants were allowed to take a short break between sessions.

In Breakout, the player controls a paddle at the bottom of the screen to bounce a ball and break bricks at the top. Hitting bricks scores points, while missing the ball results in losing one of five lives. In Space Invaders, the player controls a cannon to prevent aliens from invading. Destroying enemy ships earns points. Being hit or letting invaders reach the Earth results in the loss of one of three lives. In Enduro, the player steers a car in a racing game, earning points for successfully overtaking other cars and losing points if overtaken. Players must overtake a specified number of cars within a given time. Failing to do so results in ‘game over’. After reaching the required number of overtakes, no further points can be earned until the start of the next day.

A button box with four buttons arranged in a slightly curved row was used as controller (see Supplementary Fig. S15). The first two buttons on the left were assigned to ‘fire’ and ‘brake.’ In Enduro, the fire’ button controlled acceleration, in Space Invaders it fired the cannon, and in Breakout it launched a new ball. This button was pressed with the left middle finger. The second button, used for braking, was only functional in Enduro and was controlled by the left index finger. The two buttons on the right controlled steering: the third button, operated by the right index finger, controlled ‘left,’ and the fourth button, operated by the right middle finger, controlled ‘right.’ Combinations of these actions were possible in Space Invaders and Enduro.

In Breakout, we slowed down the paddle’s steering by applying the action taken by the participant only every second frame.

#### 2.2.3 Task implementation and data collection

The game instructions were implemented using the Tkinter library, while the Arcade Learning Environment (ALE; Bellemare et al., 2013), combined with the Python interface (Goodrich, 2015), was used to present the games and log all relevant game data. This included motor responses, received rewards, the number of episodes played per session, and the presented screens. The relevant game information was stored as a time series with a temporal resolution of 45 Hz. The observed screens were saved as sequences of matrices representing grayscale pixel intensity values. The interface was modified and adapted to match the experimental design. The experiment was presented on a 1024×768 pixel screen (width × height), with states updated at a frequency of 45 Hz. Each state consisted of the maximum pixel values from the current and previous frames to reduce flickering. States were displayed in grayscale at a resolution of 84×84 pixels, consistent with the input used for the DQNs. Only the scoreboard was shown at a higher resolution to enhance readability.

### 2.3 fMRI data aquisition and preprocessing

#### 2.3.1 fMRI aquisition parameters

The dataset was collected at the Neuroimaging Center of the TUD Dresden University of Technology. fMRI data were recorded using a 3T MRI scanner (Siemens MAGNETOM Prisma) equipped with a 32-channel head coil. Initially, a field map was acquired to correct for echo-planar imaging (EPI) distortions, with repetition time (TR) = 749 ms, short echo time (TE) = 4.92 ms, long TE = 7.38 ms, flip angle = 56°, and slice thickness = 2 mm. For whole-brain functional imaging, simultaneous multi-slice (SMS) acceleration with an SMS factor = 6 was used, with TR = 987 ms, TE = 32 ms, flip angle = 62°, FOV = 192 mm, 72 slices, and a voxel size = 2 mm isotropic. On the first day, a T1-weighted anatomical reference scan was collected with a TR = 1900 ms, TE = 2.26 ms, inversion time (TI) = 900 ms, flip = 9°, and a spatial resolution = 0.5 mm × 0.5 mm × 1.0 mm.

#### 2.3.2 fMRI preprocessing pipeline

After data acquisition, the fMRI data were preprocessed using SPM12 running on MATLAB R2021b. First, slice-time correction was applied to the functional images, considering the SMS acquisition sequence. The images were then spatially realigned and unwarped using a voxel displacement map, calculated from the field map, to correct for distortions. The high-resolution structural image of the participant was co-registered to the mean functional image and segmented. The structural and functional images were normalized to the standard Montreal Neurological Institute (MNI) space, with a resampled voxel size of 2 mm isotropic. Spatial smoothing was applied using a Gaussian kernel with a full-width at half-maximum (FWHM) of 6 mm isotropic. While real-time head motion tracking was not collected, head motion remained within a minor to moderate range for all participants, and none had to be excluded due to excessive movement. In addition to the image realignment, a separate general linear model (GLM) was calculated for motion correction, using six motion parameters (three translations and three rotations) as regressors, with the preprocessed fMRI signal as the dependent variable (Friston et al., 1996). A high-pass filter with a cutoff of 128 seconds was applied as part of the GLM specification. This procedure accounted for motion-related signal fluctuations, and the resulting residuals were used as head motion-corrected fMRI data in our analyses. The correlation maps were interpolated using trilinear interpolation for visualization.

### 2.4 Analysis

#### 2.4.1 DQN-based encoding model analysis

**Fig. 1.**
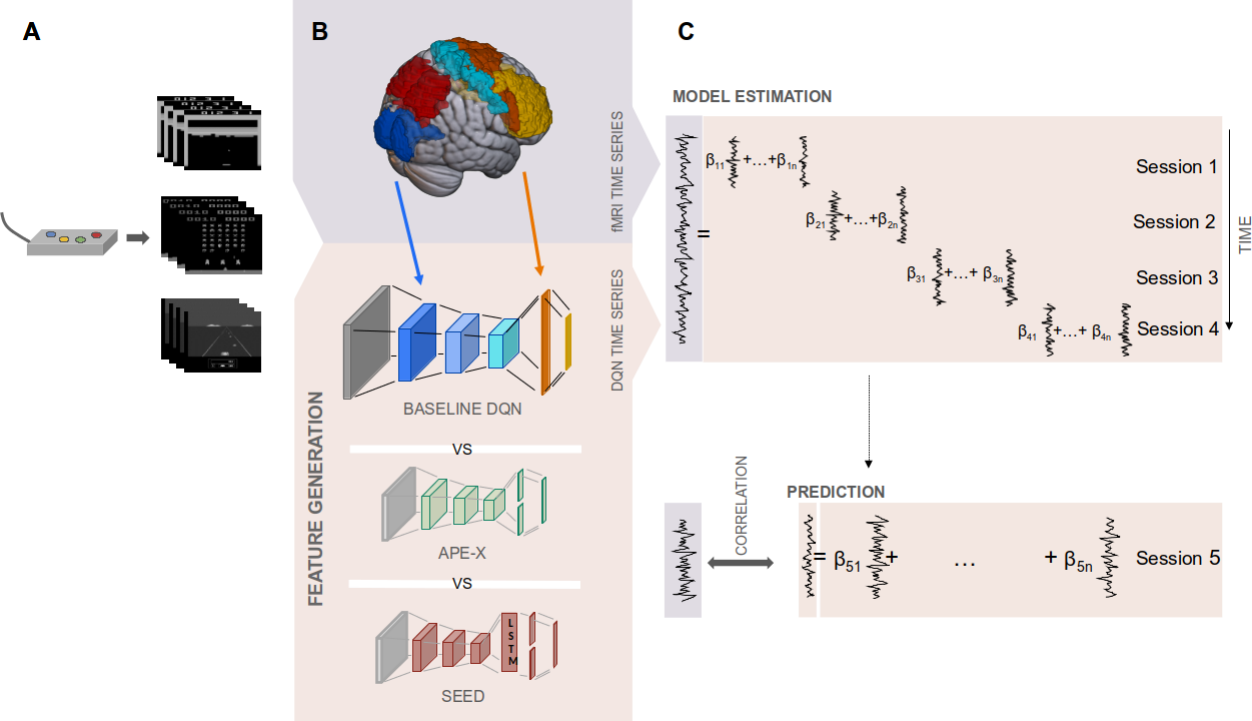
Analysis pipeline using a DQN-based encoding model. (A) Humans played arcade games inside an fMRI scanner using a four-button controller. (B) Video frames were processed through the DQNs, generating a time series of activation values for each neuron across all layers. Schematic representation of the color-coded hierarchical correspondence between DQN layers and brain regions (V1/V2 = blue, PPC = red, M1 = light blue, PMC = orange, LPFC = yellow), further analyzed in Section 3.3. The DQN comprises three convolutional layers (layers 1-3), one fully connected layer (layer 4), and an output layer. In Ape-X and SEED, the fourth layer implements a dueling architecture. In SEED, an additional LSTM layer is inserted between the third convolutional layer and the fourth layer. (C) For each game, DQN, subject, and voxel, these stimulus features served as predictors in a GLM, fitted to the voxel’s fMRI time series using four of five sessions in a 5-fold cross-validation procedure. The fitted GLM was then used to predict the fMRI time series of the held-out session, with prediction accuracy assessed via Pearson correlation between actual and predicted fMRI time series.

To model voxel activities underlying S-R transformations, we used a DQN-based encoding model approach (Güclü & van Gerven, 2015). Our encoding model analysis consisted of two components: a DQN, which nonlinearly mapped the presented stimulus into a feature space, and a GLM, which mapped these DQN-generated features into the voxel space to predict neural activity. Gameplay screens generated by the subjects were processed through a DQN that had been specifically trained on the game, resulting in a time series of activation values for each neuron across all network layers. Each entry in this time series corresponded to the activation of the neuron in response to the current input presented to the model. To predict the fMRI time series, we fitted a voxel-specific regularized GLM, which mapped the generated stimulus features onto the fMRI data. The time series of the DQN’s activation values served as predictors, while the voxel’s response served as the dependent variable (see Fig. 1). Depending on the object of investigation, the GLM predictors were selected either by including the activations of all neurons across all layers of the DQN (as in Sections 3.1 and 3.2) or layer-wise, using only the time series of neurons from a specific layer (as in Sections 3.2 and 3.3). We evaluated prediction accuracy using a 5-fold cross-validation procedure. The GLM was fitted to data from four out of five sessions. Then, we averaged the resulting regression coefficients and used them, together with the DQN-derived predictors from the held-out session, to predict the voxel’s time series in that session. The prediction performance was quantified by computing the Pearson correlation between the predicted and the actual fMRI time series. This procedure was repeated such that each session served once as the test set, while the remaining sessions were used for fitting the GLM. We compared the prediction accuracy of three encoding models, differing in the DQN component, using data from three games.

#### 2.4.2 DQN component of the encoding model analysis

DQNs are DNNs that combine deep learning with a model-free RL technique to solve complex tasks in environments with large state spaces, such as arcade games (Mnih et al., 2015). The Q-learning algorithm is used to approximate the Q-value function, where the Q-value represents the expected discounted future reward for each (state, action)-pair based on the rewards obtained from executing an action in the given state (Watkins & Dayan, 1992). The network takes the state as input and outputs the Q-values for all possible actions, and the action with the highest Q-value is then selected to solve the task.

In our encoding model analysis, the DQNs served as non-linear, feature-generating mappings. We investigated three different DQNs, each representing a distinct stage of development and complexity. In our study, we used the baseline DQN as our baseline (Mnih et al., 2015). This architecture represents a basic implementation of DQNs. Ape-X and SEED (Espeholt et al., 2019; Horgan et al., 2018) build upon the baseline, incorporating advanced training procedures and architectural improvements to enhance performance and training efficiency. SEED, the most advanced of the three models, extends Ape-X further through additional optimizations. All three DQNs share a common structure, starting with three convolutional layers with rectified linear (ReLU) activations. The first convolutional layer contains 20×20×32 units, followed by a second layer with 9×9×64 units, and a third layer with 7×7×64 units. Each DQN includes an output layer whose neurons correspond to the possible actions. At each time step, the DQN received a stack of four consecutive video frames as input and information on ‘game over’ states. For detailed information on the preprocessing of input frames, we refer to Haberland et al. (2025). A visualization of gameplay for the baseline DQN and Ape-X is available on our GitHub repository https://github.com/SHaberland15/Arcade_DQN_Research_fMRI/tree/main/media. Here, we provide only a brief overview.

For training curves, see Supplementary Figure S14. Table 2 shows the gaming performance of the three DQNs in comparison with human performance. An illustrative comparison of human and DQN gameplay behavior is provided in the GitHub repository at https://github.com/SHaberland15/Arcade_DQN_Research_fMRI.

#### 2.4.3 GLM component of the encoding model analysis

After generating the stimulus features and preprocessing the fMRI data, we used voxel-specific regularized GLMs as linear encoding models. These models were fitted on training data to predict voxel activity in the test set across each DQN, game, and participant. The selection of predictors, whether layer-specific or derived from all layers, was determined by the research objectives. Only the time series of neurons that showed activation at least once during feature generation were included as predictors. We used activations from the convolutional layers following the application of the ReLU activation function. The number of predictors in the design matrix varied depending on the layer, game, DQN model, and subject. On average, the layers contributed approximately: ∼10,000 for the first, ∼5,000 for the second, ∼3,000 for the third, and ∼500 for the fourth layer. In SEED, the LSTM layer contributed additional ∼500 predictors prior to any regularization (see Section 2.4.2 for layer details). The design matrix, composed of these neuronal time series, was standardized along the temporal and feature dimensions by converting them into z-scores. To account for the hemodynamic delay, the regressors were convolved with a hemodynamic response function (HRF). To match the generated features with the fMRI sampling rate (TR frame rate), predictors were downsampled to the EPI frequency and a high-pass filter with a cutoff of 128 seconds was applied. Before solving the GLM, the predictors and the fMRI time series were z-scored again along the temporal dimension. Because the number of regressors exceeded the number of available data points (except in GLMs restricted to output layer neurons), it was necessary to regularize the GLM during the training phase of the 5-fold cross-validation, extending the standard GLM to a regularized GLM. In this context, L1-regularization proved the most effective (Mohr & Ruge, 2021). Unlike unregularized or L2-regularized models, L1-regularization requires iterative solving. The model was solved using the MATLAB toolbox lasso_gpu (Mohr & Ruge, 2021). For each voxel, we computed the cross-validated Pearson correlation across a range of regularization parameters λ∈[0.005,0.5] and observed an approximate performance plateau in the mean correlation, averaged across all subjects and voxels within the ROIs, in the range λ∈[0.04,0.1] . We therefore fixed the midpoint of this interval as the regularization parameter for all voxels. Voxel responses in the test set were predicted using a linear combination of the test-set features and weighted by the mean regression coefficients estimated from the training folds. The predicted voxel time series was then compared to the measured voxel time series using Pearson correlation. For each voxel, prediction accuracy was calculated as the average Pearson correlation across participants, games, and test sessions, separately for each DQN-based encoding model. For the second-level analysis, the individual correlation maps were first Fisher Z-transformed. Voxel-wise one-sample t-tests were then performed in SPM across subjects, and the resulting t-maps were subjected to family-wise error (FWE) correction (p < 0.05). We normalized the confidence intervals according to Morey (2008).

#### 2.4.4 Selection of regions of interest

To investigate brain activity along the dorsal stream, we defined a set of regions of interest (ROIs). The early visual ROI was based on the Julich Brain Atlas and included regions V1 and V2. The posterior parietal cortex (PPC) was defined using the AAL2 atlas and included the precuneus and superior parietal lobule (SPL). The primary motor cortex (M1) was defined anatomically as Brodmann area 4 (BA 4), and the premotor cortex (PMC), including the supplementary motor area (SMA), corresponded to Brodmann area 6 (BA 6). The lateral prefrontal cortex (LPFC) included the middle frontal gyrus, inferior frontal gyrus pars triangularis, and pars opercularis, also based on the AAL2 atlas. The dorsal ROI encompassed the PPC, M1, and PMC. To enable comparison with the ventral visual stream, the ventral ROI included the inferior temporal cortex (IT) and middle temporal gyrus (MTG), again based on the AAL2 atlas. All ROI masks were created using the WFU PickAtlas toolbox in SPM12.

### 2.5 Software and hardware

The experiment and analysis were conducted on Ubuntu 20.04 as the operating system (see also Section 2.2.3). The experiment was run within a Docker container based on the python:3.9.7-buster image, which included Python version 3.9.7 and was built on Debian 10 (Buster). The DQNs were trained and used for feature extraction within Docker containers using the ALE framework (Bellemare et al., 2013) and its Python interface (Goodrich, 2015). The baseline DQN and Ape-X were trained on Debian ‘Buster’ using Python 3.8.8 and PyTorch. SEED was trained with Python 3.6.9 and TensorFlow, running on Ubuntu 18.04.5 LTS. All analyses and estimations of the regularized linear models were performed in MATLAB 2019a. Our computational setup included an NVIDIA RTX A4000 GPU with 16 GB of memory and an Intel Xeon W-2245 CPU with 8 cores and 64 GB of RAM.

## 3 Results

To investigate S-R transformations, we analyzed fMRI data from N=23 human participants while they played the arcade games Breakout, Space Invaders, and Enduro. To predict voxel activity, we used a DQN-based encoding model approach (see Fig. 1). A DQN was used as a feature-generating mapping, processing the video screens seen by the subjects through the DQN to generate time series of activation values for each neuron in each layer. Second, we implemented a GLM as our encoding framework, using the generated stimulus features as predictors and the time series of voxel activations as the dependent variable. Within a 5-fold cross-validation procedure, we fitted these features to the measured fMRI time series during the training phase. We evaluated their prediction accuracy during the test phase by calculating the Pearson correlation ρ between the actual fMRI time series and those predicted by the GLM for each voxel. To assess whether advancements in RL could improve the accuracy of modeling voxel activity, we compared the prediction accuracy of three DQN-based encoding models, each differing in their feature-generating component. We evaluated the predictive performance for each subject on three arcade games on which the DQNs were trained without any human data. To ensure generalizability beyond a single task, we averaged the results across all subjects and the three games.

### 3.1 DQN-based encoding models predict voxel activity of task-related S-R transformations across the dorsal stream

**Fig. 2.**
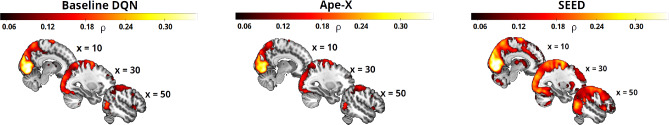
Prediction accuracy of the three encoding models. The plots show whole-brain Pearson correlations between the actual and predicted fMRI voxel time series for the three encoding models, using feature maps from the baseline DQN (left), Ape-X (middle), and SEED (right). Neurons from all layers of the DQNs were used as predictors. High prediction accuracies were primarily observed in regions along the dorsal stream. Only statistically significant voxels are shown (FWE-corrected, p < 0.05).

To localize brain regions involved in transforming time-continuous visual stimuli into motor responses, we applied the encoding models using the activations from all layers of the DQN as predictors in the GLM. All three encoding models yielded significant predictions of voxel activity along the dorsal visual stream (FWE-corrected, p < 0.05). All models were able to capture brain activity in the early visual cortex, progressing through higher-order visual areas, and extending into the PPC and further into motor-related regions. In the frontal cortex, activations covered the precentral gyrus and the SMA, including M1 and PMC. Activations also extended into the LPFC (see Fig. 2 and see Supplementary Tables S11–S13 for activation peaks). For completeness, Supplementary Figure S1 shows the results separately for each of the three games.

A subsequent analysis comparing the dorsal with the ventral visual stream showed that all three encoding models predicted voxel activity significantly higher in the dorsal ROI (precuneus, SPL, M1, and PMC) compared to the ventral ROI (MTG, IT) (two-sample t-test, p < 0.001, see Supplementary Fig. S17), which is consistent with the well-established hypothesis that visuomotor tasks are predominantly associated with the dorsal stream rather than the ventral stream (Goodale, 2011; Kravitz et al., 2011). While these results align with theoretical expectations, it should be noted that potential differences in ROI characteristics, such as size and signal quality, may limit direct comparability between dorsal and ventral regions.

These results provided initial validation that DQN-based encoding models enable the modeling of time-continuous S-R transformations using high-dimensional stimuli in biologically plausible brain regions. They offer preliminary evidence for similarities between the internal feature representational spaces of DQNs and those of the human brain, motivating a more detailed layer-wise analysis to characterize the step-wise S-R transformation process.

### 3.2 Features from advanced DQNs enable more accurate predictions of voxel activity

**Fig. 3.**
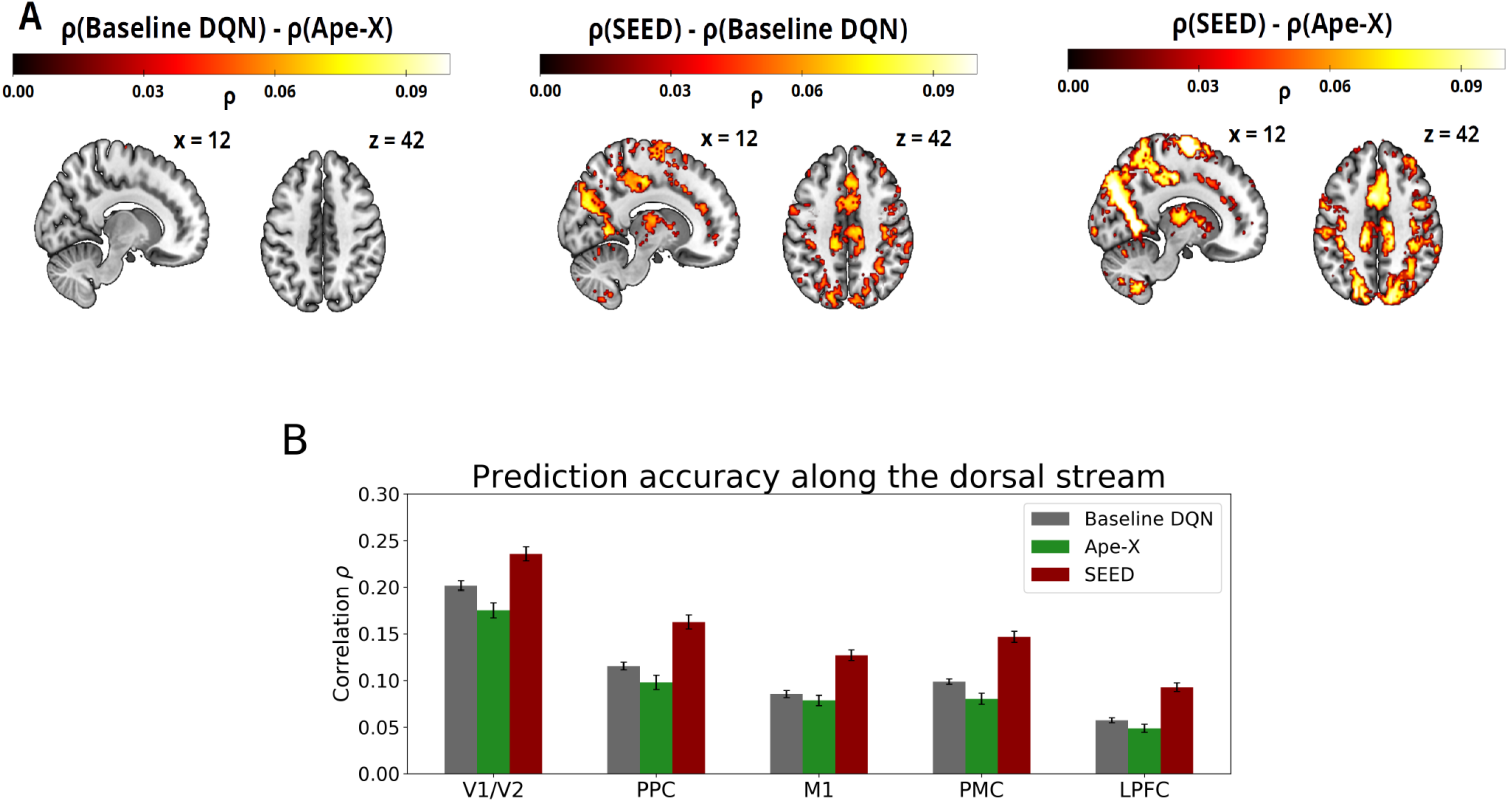
(A) Differences in prediction accuracy between the three encoding models. Intensity values reflect significant differences in voxel-wise Pearson correlations, highlighting regions where the feature representations of one DQN outperform those of another in predicting voxel activity (FWE-corrected, p < 0.05). No considerable differences were observed between Ape-X and the baseline DQN (left). SEED outperformed the baseline DQN (middle) and Ape-X (right). Features from all network layers were used as predictors in the encoding models. Reverse subtractions revealed no voxels with significant positive differences (FWE-corrected, p < 0.05). SEED generated the most effective feature representations for predicting voxel activity, showing the greatest improvements in voxels within the PPC and PMC regions. (B) Prediction accuracy of the three encoding models across ROIs. Bar plots show voxel-wise Pearson correlations within each ROI. Encoding models were based on features from the baseline DQN (gray), Ape-X (green), and SEED (red). The ROIs encompass V1/V2, PPC (precuneus, SPL), M1 (BA 4), PMC (BA 6), and LPFC (middle frontal gyrus, the inferior frontal gyrus pars triangularis, and pars opercularis). Error bars represent the normalized 95% confidence interval of the mean correlation across subjects. The improved prediction based on features generated by SEED was particularly evident in higher-level visual and control regions.

We demonstrated that the feature representations of all three DQNs are well-suited for predicting neural activity along the dorsal visual stream during gameplay. By comparing the encoding performance of the three DQNs and their strengths and limitations, we assessed the extent to which recent advances in machine learning contribute to improvements in neuroscientific modeling, potentially offering insights into the representational structure and computational principles of the human brain. We analyzed the differences in prediction accuracy across the three encoding models and subsequently investigated the origins of these differences. Our analysis revealed that SEED was the most effective feature-generating mapping, significantly outperforming the baseline DQN and Ape-X in terms of prediction accuracy along the dorsal stream (FWE-corrected, p < 0.05, see Fig. 3A and see Supplementary Tables S14–S16 for activation peaks). Differences in predictions between the DQNs were observed in all investigated ROIs (repeated-measures ANOVA with the factors ROI and DQN , Greenhouse-Geisser corrected, revealed significantly main effects of ROI: $F_{\text{ROI}}(2.23)=129.34$ , $\eta_{p}^{2}=$ 0.86 , and DQN: $F_{\text{DQN}}(1.42)=156.62$ , $\eta_{p}^{2}=0.88$ , and a significant ROI×DQN interaction: $F_{\text{ROI}\times \text{DQN}}(4.78)=17.87$ , $\eta_{p}^{2}=0.46$ , all p < 0.001; post hoc comparisons indicated that, within each ROI, SEED achieved significantly higher prediction accuracy than the baseline DQN and Ape-X, Bonferroni-corrected, p < 0.001, see Fig. 3B). These differences in prediction accuracy between the features of SEED and the other DQNs were particularly pronounced in higher-level visual and control regions, such as the PPC and PMC and less pronounced in lower-level areas, including V1/V2 and M1 (custom contrasts, Bonferroni-corrected, all p < 0.05, except PPC vs. V1/V2, after Bonferroni-correction p=0.092 , see Supplementary Tables S4 and S5). No considerable differences in prediction accuracy were observed between the features of Ape-X and the baseline DQN, with only a few voxels reaching statistical significance (FWE-corrected, p < 0.05, see Fig. 3A left). While all three DQNs were able to generate features that predict voxel activity in lower-level regions, higher-level regions appear to require more abstract or complex features, captured better by the most advanced model, SEED.

**Fig. 4.**
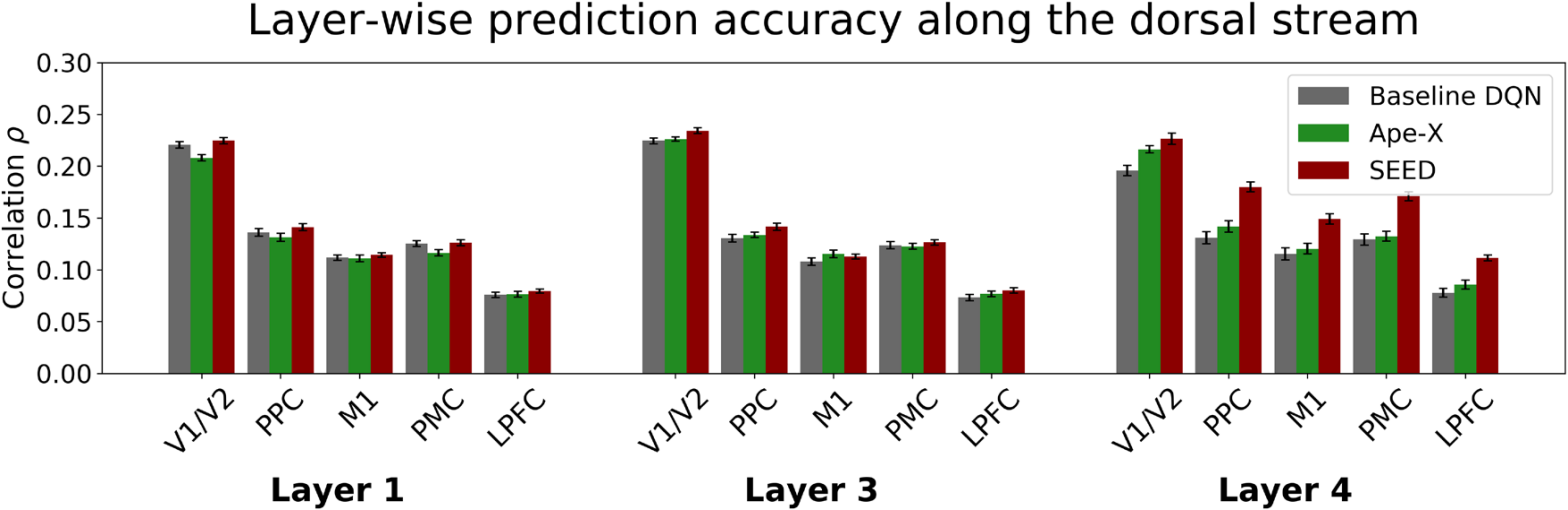
Layer-wise comparison of prediction accuracy. The bar plot shows the Pearson correlation between actual and predicted time series for individual layers for voxels within V1/V2, PPC, M1, PMC, and LPFC. The analysis included DQN neurons from the first, third, and fourth layers, using encoding models based on features from the baseline DQN (gray), Ape-X (green), and SEED (red). Error bars represent the normalized 95% confidence interval of the mean correlation across subjects. Breaking down predictive performance by layer enabled a direct comparison of representational alignment between models and brain regions, offering insights into which stages of computation most closely resemble neural processing. While the convolutional layers share the same architecture across all DQNs, architectural differences, particularly in the fourth layer, were reflected in prediction accuracy. Notably, SEED’s fourth layer generated features that better explain voxel activity, particularly in higher-level control regions, than those of the baseline DQN and Ape-X.

After demonstrating that advances in RL can be leveraged to improve modeling in neuroscientific research, we performed a layer-wise breakdown and comparison of the DQNs’ encoding capabilities. This allowed us to identify the hidden network layers that contributed most substantially to the observed differences and to gain insight into the functional role of each computational stage. We addressed this by replacing the GLM, which included neural activity from all DQN layers as predictors, with voxel-specific GLMs incorporating layer-specific predictors (see Fig. 4 and Supplementary Fig. S2). All three DQNs shared an identical architecture in their initial layers, consisting of three convolutional layers. As the three convolutional layers within each individual DQN tended to show similar patterns, we focused our analysis on layers 1 and 3 as representatives of the three convolutional layers to maintain clarity. Because the architectures diverge between the DQNs after the convolutional layers, the representations learned in the early layers may already differ. Although significant differences between the DQNs emerged already in the earlier layers (repeated-measures ANOVA for the first and third layer with the factors DQN , Layer and ROI , Greenhouse-Geisser corrected, revealed a significantly main effect of DQN: $F_{\text{DQN}}(1.91)=10.32$ , $\eta_{p}^{2}=0.33$ , and a significant Layer×DQN interaction: $F_{\text{Layer}\times \text{DQN}}(2.00)=11.68$ , $\eta_{p}^{2}=0.36$ , all p < 0.001), the magnitude of these differences remained small across the first and third layer in all examined ROIs. In contrast to the first convolutional layers, the fourth layer showed significantly larger differences between the DQNs in prediction accuracy across the ROIs (custom contrast of the DQN×Layer interaction, p < 0.001, see Supplementary Tables S6 and S7). This suggests that the early convolutional layers of the DQNs, despite differences in training regimes and architectural complexity, contribute to prediction to a similar extent, with the differences shown in Figure 3 being primarily attributable to SEED’s fourth layer, the layer in which architectural divergence becomes prominent. Differences in prediction accuracy between features generated by SEED’s fourth layer, which follows the LSTM, and those from the fourth layers of the baseline DQN and Ape-X were more pronounced in higher-level control regions, including the PPC and PMC, than in lower-level areas such as V1/V2 (custom contrasts, Bonferroni-corrected, p < 0.01, see Supplementary Tables S8 and S9, and see Supplementary Figures S7 and S8). This pattern suggests a potential functional relevance of the LSTM component in capturing representations related to higher-level cognitive control processes.

### 3.3 Advanced DQNs capture brain-like hierarchical processing stages

**Fig. 5.**
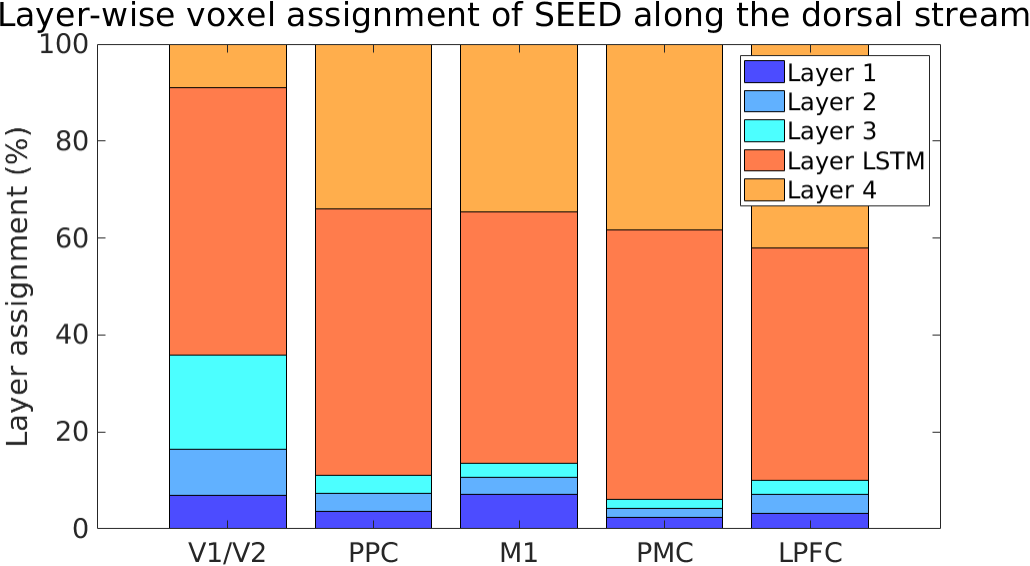
Voxel assignment to the layer of maximal prediction accuracy in SEED. To identify which hidden layers of SEED provided the most predictive features for different brain regions, each significant voxel (FWE-corrected, p < 0.05) was assigned to the hidden layer yielding the highest layer-wise correlation between predicted and actual activity. The proportion of each layer in the bars (per ROI) reflects the percentage of voxels within that ROI assigned to the corresponding layer. This analysis was conducted for V1/V2, PPC, M1, PMC, and LPFC, following the approach in (Güclü & van Gerven, 2015, 2017a). In V1/V2 and M1, the proportion of voxels predicted by the first convolutional layers was larger than in the other ROIs. In higher-level areas, such as PPC, PMC, and LPFC, the proportion of voxels associated with early layers progressively decreased, while the predictive contribution of the later layers increased. A strict one-to-one mapping between network layers and brain regions is unlikely because of the dependent nature of the network, where the layer’s output serves as input to the next.

Since SEED has proven to be a suitable feature-generating mapping for predicting voxel activity along the dorsal stream, we focused our further analysis on SEED to investigate whether it captures the different stages of human visuomotor processing. Understanding how SEED’s internal representations align with the brain’s processing hierarchy can deepen our understanding of neural coding and may help characterize the functional organization of neural computations in this stream from a computational standpoint. To assess whether the hierarchical structure of SEED aligns with the organization of the dorsal visual stream, we compared the voxel-wise prediction accuracy by assigning each voxel to the SEED layer that yielded the highest layer-wise prediction accuracy. This voxel-wise mapping revealed that most voxels best predicted by the three early convolutional layers were located in V1/V2 and M1 (see Fig. 5). In the low-level visual areas V1/V2, the ratio of early (first, second, and third) to late (LSTM and fourth) layers was significantly higher than in the higher-level ROIs, whereas the ratio decreased in regions such as the PPC, PMC, and LPFC (pairwise t-tests for the factor ROI on the ratio of early layers to late layers, after applying the log-ratio transformation described in (Greenacre, 2021) to account for compositional data, p < 0.001, Bonferroni-corrected, see Supplementary Table S10). These results suggest that SEED captures brain-like hierarchical representations, with its layer-wise representations reflecting the cortical progression from early visual to higher-order visuomotor areas along the dorsal visual stream (see also Supplementary Fig. S4).

## 4 Discussion

Understanding how the human brain continuously transforms complex, high-dimensional sensory inputs into motor responses within a dynamically changing environment is crucial for explaining everyday behavior. To gain a more detailed characterization of the neural representations underlying individual S-R processing stages, we analyzed human gameplay behavior during arcade games performed inside an fMRI scanner, using a DQN-based encoding model. We demonstrated similarities in internal representational feature spaces between artificial and biological neural networks. By comparing three DQNs with varying architectures and training methods, we showed that recent advances in machine learning can be leveraged to model aspects of S-R processing. We also found analogies to hierarchical information processing in the brain.

While encoding models have been widely used to model neural brain activity, the rapid evolution of deep learning methods has opened new possibilities for neuroscience. The integration of DNNs in encoding models enabled the characterization of the visual cortex using hierarchically organized object-recognition CNNs (Cichy et al., 2016; Yamins & Dicarlo, 2016). Nevertheless, capturing task-relevant brain activity in naturalistic, interactive environments remains challenging.

Video games offer a promising framework to address this issue: they are time-continuous, require active engagement, and involve a wide range of cognitive processes rather than isolated functions (Bellec & Boyle, 2019). However, the video game paradigm has received limited attention in the literature so far (Cross et al., 2021; Kemtur, 2023; Paugam, 2024; Tomov et al., 2023). These studies have shown that RL-trained models not only acquire task-solving skills but also develop effective encoding capabilities. Therefore, we leveraged a DQN-based encoding model applied to Atari gameplay. From a cognitive neuroscience perspective, Atari games are particularly well-suited for this purpose, as they enable the study of S-R transformations within a controlled yet high-dimensional and time-continuous environment. By engaging multiple visuomotor processes, they provide an appropriate framework for investigating the dorsal visual stream (Bellec & Boyle, 2019; Goodale & Milner, 1992). From a technical perspective, Atari games are also widely regarded as benchmarking environments for RL models. Although they offer greater ecological validity than conventional experimental tasks, they remain relatively simple. This simplicity reflects practical and computational constraints of the present study rather than a fundamental limitation of deep RL approaches. However, this relative simplicity should not be equated with triviality. For human players, these environments are far from exhaustively predictable. All three games operate in high-dimensional state spaces and are initialized with random seeds, preventing fully deterministic gameplay. Consequently, successful performance requires continuous visuomotor integration, rapid action selection, and ongoing adaptation to dynamically changing and unexpected game states rather than the execution of fixed routines. Overall, Atari games strike a pragmatic balance between ecological relevance and experimental feasibility.

However, playing arcade games may engage more than simple S-R transformations. High-level cognitive processes may be involved, and behavior may appear goal-directed and anticipatory while playing these games. Since participants were not required to have prior video game experience and to increase the likelihood of observing more automated processing, participants practiced the games prior to scanning to reach a stable performance level (Mohr et al., 2016). On average, participants achieved performance levels comparable to the expert scores reported by Mnih et al. (2015) (see Table 2). We further observed no relationship between individual gaming performance and encoding model performance, suggesting that differences in gaming skill do not account for the DQN-based prediction of neural activity (see Supplementary Fig. S16).

Our findings support previous evidence that DQNs, when used as feature-generating mappings, enable the investigation of S-R transformations along the dorsal stream in time-continuous and complex environments. Our results are consistent with those reported in Cross et al. (2021), although the predictions of our baseline DQN were, on average, lower than those of some individual subjects in Cross et al. (2021), partly due to inter-subject variability. Moreover, these differences may also arise from methodological variations, ranging from preprocessing steps to the choice of hyperparameters, which we observed to have a notable impact on the results. While these factors do not affect the relative comparisons between the DQNs, which remain consistent across analyses, they highlight the model’s sensitivity to such analytical choices.

To ensure that the predictive performance of the DQN-based models is not solely driven by the encoding of low-level visual features, we employed a control model based on a linear transformation of the raw pixel values of the observed screens (see Supplementary Fig. S12). Additionally, we implemented a second control model using the motor responses of the human participants as predictors to assess whether the neural variance captured by the DQNs extends beyond that explained by motor responses (see Supplementary Fig. S13). Features derived from the DQNs showed clear advantages over both control models in prediction accuracy across visual and motor ROIs (FWE-corrected, p<0.05 ), with only a small cluster of the motor cortex showing slightly higher performance for the motor-response control model compared to the baseline DQN and Ape-X models. These findings indicate that modeling neural activity in certain regions requires more than linear transformations of sensory input or behavioral motor output, and instead benefits from more complex and abstract representations, such as those learned by the DQN. Moreover, the architecture alone was not sufficient to predict brain activity (see Supplementary Fig. S9), and Cross et al. (2021) further demonstrated that neither a DQN trained on a different game nor a variational autoencoder can match the performance of a task-trained baseline DQN. Together, these results underscore the importance of task-specific learning for effective encoding of neural responses.

The comparison of different training objectives also highlights the importance of RL in shaping model performance. When controlling for data quantity and type, RL outperforms imitation learning, which models human gameplay (Kemtur, 2023), and vision models in encoding brain activity during video gameplay (Paugam, 2024).

In this study, we focused on three different DQNs that shared the same training objective, maximizing reward through RL, but differed in training methods and architectural complexity. By comparing these models, it revealed their respective strengths and weaknesses as feature-generating mappings and identified key factors contributing to successful neural encoding. This, in turn, improves the ability to model neural mechanisms, which is essential for advancing the understanding of brain function (Cichy et al., 2016; Kriegeskorte, 2015). At the behavioral level, it has been shown that increased model complexity is associated with higher predictive accuracy of human motor responses (Haberland et al., 2025). Although improvements in encoding performance were observable behaviorally, more pronounced and varied advantages were observed at the neural level, as reflected by the increasing similarity of stimulus representations across brain regions. When comparing the predictive performance of the features across the three DQNs at the neural level, we found that the features from SEED, the most advanced of the three, outperformed those from the other DQNs. Differences emerged particularly in ROIs associated with higher-level cognitive control functions, such as the PPC and the PMC (Brovelli et al., 2015; Bunge et al., 2002; Podzebenko et al., 2002). In contrast, differences in prediction accuracy across the models were small in low-level areas, including V1/V2 and M1. This may indicate that all three models capture aspects of low-level sensory information to a comparable extent. The layer-wise analysis revealed that the first three convolutional layers of the DQNs generated features yielding comparable prediction accuracies across ROIs. This confirms the assumption that differences in prediction performance do not arise from differences in the representation of low-level sensory stimulus features, but only become relevant at higher levels of abstraction (Güclü & van Gerven, 2015). In contrast, substantial differences emerged in the fourth layer, where SEED outperformed the baseline DQN and Ape-X, which both showed limited encoding performance in higher-level visual and control regions (see also Supplementary Figs. S7 and S8). These findings indicate that SEED’s higher layers develop more task-specific and architecture-sensitive representations, which are crucial for modeling brain activity in regions associated with higher-level cognitive control. This advantage is likely attributable to SEED’s architectural enhancements, specifically, the incorporation of an LSTM module, whose output is the input of the fourth layer, and the dueling network architecture.

LSTMs are RNNs that take temporal dependencies in data into account (Hochreiter & Schmidhuber, 1997). They filter task-relevant content using a system of gates, similar to mechanisms hypothesized for human working memory (Chatham & Badre, 2015; Frank et al., 2001). As a result, LSTMs are increasingly used as models to investigate working memory (Goldstein et al., 2019; Sainath, 2020). Behavioral evidence suggests that incorporating an LSTM module is an important advancement in modeling human gameplay (Haberland et al., 2025). Our study suggests that temporally integrated representations of the LSTM improve the model’s sensitivity to temporal dynamics, improving its ability to capture brain-related features in higher-level regions, extending up to the LPFC (see also Supplementary Figs. S7 and S8). These areas are typically associated with cognitive control functions such as working memory maintenance and decision making (Smith & Jonides, 1999; Wager & Smith, 2003). However, compared to other regions, the correspondence between DQN-generated features and activity in the dorsolateral prefrontal cortex (dlPFC), a region typically associated with more abstract forms of cognitive control and the manipulation of working memory contents, was less pronounced (D’Esposito et al., 1999). This could reflect the relatively low demands on complex memory operations in Atari gameplay, which is largely stimulus-driven, or it may indicate limitations in the ability to capture such high-level cognitive functions with our models. Nonetheless, the observed patterns suggest that the LSTM layer may encode brain-relevant features of visual stimuli that align with the functional roles of these regions, highlighting the importance of recurrent connections for modeling brain activity in a time-continuous task. This is consistent with findings from cognitive science, which indicate that the human brain is a highly dynamic system with extensive recurrent connectivity, providing a strong theoretical motivation for incorporating such components (Indiveri & Liu, 2015; Kriegeskorte, 2015; Lamme & Roelfsema, 2000)

However, comparisons based on encoding model performance must account for potential confounding factors. For example, SEED contains more hidden units than the baseline DQN and Ape-X due to the implementation of an LSTM, including an additional layer with 512 hidden units that connects the convolutional layers to the LSTM. While a larger number of units does not necessarily lead to performance improvements, it nevertheless represents a potential confound that should be considered (Nonaka et al., 2021). Beyond model architecture, encoding performance is also substantially influenced by DNNs’ task performance (Schrimpf et al., 2018), their training dataset, and the amount of training (Paugam, 2024). In our study, such differences in training of the DQNs could act as potential confounds, and the observed effects may, at least in part, reflect differences in training experience in addition to architectural characteristics. However, training was terminated only once no further improvement in gaming performance was observed, suggesting that differences in the amount of training data across the DQNs may have had a limited impact on the primary results of our research question. At the behavioral level, it was shown that encoding performance depends on gaming performance rather than on the amount of training data (Haberland et al., 2025). Nevertheless, this represents a potential source of bias that should be taken into account when interpreting the results. In our analysis, the fitting of the encoding components was always performed in the same way and on the same fMRI data across all three DQNs.

The layer-wise analysis across different DQNs not only highlights the importance of recurrent structures but also reveals additional insights through the functional hierarchical correspondence between SEED’s network layers and the stages of S-R transformations along the dorsal stream (see also Supplementary Figs. S3 and S4). Early layers showed stronger alignment with the early visual cortex and, to a lesser extent, with M1, whereas higher-order regions such as the PPC and PMC aligned more closely with later layers (see Fig. 5). This suggests that different brain regions are best characterized by distinct underlying feature spaces of varying complexity. Importantly, this highlights the role of multiple stages of nonlinear feature transformations in capturing higher-order cognitive processes (Bengio, 2009; Creem-Regehr, 2009; Güclü & van Gerven, 2017a). Previous studies on video gameplay using DQNs without recurrent components found no evidence of a hierarchical gradient (Cross et al., 2021; Paugam, 2024). However, an imitation learning model that incorporated recurrent components showed hierarchical organization in video game tasks (Kemtur, 2023), supporting the idea that recurrence enables temporal dynamics to be captured and may be critical for hierarchical gradients. Moreover, this functional correspondence between artificial and biological hierarchies provides a promising basis for future studies investigating how different brain regions contribute to visuomotor transformations and how these computations are internally represented. Similar hierarchical correspondences have been observed in previous work on object recognition along the ventral (Eickenberg et al., 2017; Güclü & van Gerven, 2015; Nonaka et al., 2021) and dorsal (Cichy et al., 2016; Güclü & van Gerven, 2017a) visual streams. In our study, the convolutional layers did not show a hierarchy within the visual cortex, potentially reflecting discrepancies in task demands or architectural constraints (see Supplementary Fig. S5). Prior research has also suggested that high-performing object recognition DNNs are not necessarily more brain-like (Nonaka et al., 2021; Schrimpf et al., 2018). Among the models we tested, SEED achieved the highest task and encoding model performance and showed the most pronounced representational gradient (see Supplementary Fig. S3). This raises the question of whether a similar trade-off exists in more advanced DNNs trained on S-R transformation tasks, specifically, whether increasing task performance might come at the cost of human-like encoding.

Although the DQNs captured suitable representational spaces, they generated a large number of features in each layer, resulting in a high number of GLM regressors compared to the limited number of data points, which made the models susceptible to the curse of dimensionality (Zahavy et al., 2016). To address this issue and make the estimation of the encoding models feasible, L1-regularization was applied (Ayinde & Inanc, 2019b; Jia et al., 2022; Mohr & Ruge, 2021; Mohr et al., 2015). Regularization substantially reduced multicollinearity among the GLM regressors, although some correlations within and across layers remained (see Supplementary Tables S1–S3), which can influence GLM parameter estimates (Monti, 2011). Including features from all layers could, therefore, increase model complexity without necessarily improving predictive performance over layer-wise models (see Supplementary Fig. S10). However, strong regularization removed even important information, leading to a lower encoding performance. Reducing feature redundancy during DQN training could, thus, be a useful strategy, and several methods exist to quantify and mitigate this issue (Ayinde & Inanc, 2019a; Jia et al., 2022).

While these linear encoding models are data-efficient, easy to implement, and provide interpretable mappings between feature and brain activity spaces, further improvements in encoding could be achieved using nonlinear approaches such as recurrent networks (Güclü & van Gerven, 2017b; Zhang et al., 2019).

Our results contribute to the broader debate about whether DNNs are suitable models of cognitive processes, emphasizing the importance of critically evaluating their limitations while recognizing their potential. DQNs can be regarded as an extension of RL models, which have been linked to reward-based learning mechanisms in the brain. However, the development of DNNs is typically driven by engineering objectives rather than neurobiological theories, which limits their relevance as models of brain function (Kriegeskorte, 2015; Lake et al., 2017; Ma & Peters, 2020; Marblestone et al., 2016; Yamins & Dicarlo, 2016). This also contributes to their limited ability to generalize across tasks and also in capturing neural processes in new situations (Paugam, 2024). Furthermore, critics argue that DNNs provide limited explanatory power for cognitive processes, as their internal processes are often opaque and difficult to interpret (Cichy & Kaiser, 2019). Moreover, although DNNs can achieve high predictive accuracy when used as feature-generating mappings in encoding models, such performance does not imply that they capture the complexity of the brain’s functional mechanisms or the underlying biological processes. Our analysis showed that DQN-based encoding models were able not only to predict neural activity but also to predict human motor responses with high accuracy using only the calculated Q-values of the DQNs, suggesting that these models capture task-relevant representations that extend from neural activity to behaviorally relevant mechanisms (see Supplementary Fig. S11) (Haberland et al., 2025; Mohr et al., 2019). However, in the present study, the ability of DQNs to model human behavior does not necessarily translate into better predictions of neural activity at the individual level (see Supplementary Fig. S6). This suggests that, while a DQN may capture aspects of behaviorally relevant processing, its predictive success should be interpreted with caution. A clear distinction between representational correspondence and mechanistic interpretability is, therefore, crucial (Shmueli, 2011; van Gerven, 2017). Against this critical background, however, they remain the most practical and general class of models, which are capable of learning to map high-dimensional input to appropriate motor responses, with initial evidence suggesting similarities in their representational spaces with the human brain, supporting the interpretation that these features reflect task-relevant representational structure aligned with information in the neural signals. In addition to encoding models, other successful approaches exist for investigating S-R transformations in the brain using features generated by DNNs (Doerig et al., 2023; Kriegeskorte & Douglas, 2018). At the behavioral level, DNN outputs can be directly compared with human responses (Doerig et al., 2023; Haberland et al., 2025; Mohr et al., 2019). At the neural level, decoding-based approaches aim to predict stimulus features from brain activity evoked by those stimuli. While decoding approaches primarily address whether specific information is present in a given brain region (Cox & Savoy, 2003; Haynes, 2015; Haynes & Rees, 2006; Kriegeskorte & Douglas, 2019; Tong & Pratte, 2012), complementary methods are required to characterize how that information is structured and represented in the brain. An approach to gain insight into the relational structure of representations across models and neural data is representational similarity analysis (RSA). Although RSA does not directly predict neural activity, it provides a powerful framework for assessing similarities and differences between feature spaces across different brain regions and layers of DNNs.

The pragmatic value of DNNs in cognitive neuroscience lies in their ability to scale to more ecologically valid tasks that are often inaccessible to traditional analytical models, and to adapt flexibly to new situations. With ongoing improvements in interpretability and by implementing human-inspired mechanisms, DNNs have the potential to serve as useful tools for testing hypotheses (Yamins & Dicarlo, 2016) and designing optimal stimuli (Bashivan et al., 2019). Importantly, this DQN-based encoding model approach is a highly exploratory approach that should be viewed as a valuable complement, not a replacement, for the traditional discrete and trial-based experimental paradigms. We argue that DNNs are valuable scientific tools. A model should not only be interpretable but must also achieve sufficient predictive performance to justify making explanatory claims (Cichy & Kaiser, 2019).

Future developments in DNNs might benefit from integrating alternative assumptions, biologically inspired constraints, and objective functions that represent various theories of brain function. This may help align artificial dynamics more closely with neural processes (Bellec & Boyle, 2019; Federer et al., 2020). Additionally, advancements like multi-task learning (Parisotto et al., 2015) and transformer models (Vaswani et al., 2017) provide promising directions for further development. Paugam (2024) proposed combining an encoding model with an autoregressive component. This approach assumes that the encoding model accounts for extrinsic, stimulus-driven variance in neural activity, while the autoregressive part captures intrinsic dynamics, such as autocorrelations in the neural signals. Complementary analytical methods, such as RSA, together with neuroimaging techniques like EEG, can further improve the understanding of the temporal correspondence between model-derived and neural representational structures within individual state-spaces. Analyzing specific game contexts can reveal latent subcomponents of behavior and reveal context-dependent S-R mappings, helping to identify which aspects of the underlying mechanisms of human action evaluation are captured by the DQNs. While more complex architectures are capable of mastering richer game environments such as Dota 2 (Berner et al., 2019), investigating these settings currently requires substantial computational and methodological resources. Nonetheless, such environments provide a broad range of stimuli and, combined with ongoing advances in machine learning, open entirely new avenues for future cognitive neuroscientific research. While our primary goal was to model S-R processing using brain-inspired artificial networks, our findings may also contribute to the development of more cognitively informed and efficient machine learning models.

Although challenges remain, our results provide additional proof of concept for a promising approach to investigate the neural representations underlying S-R transformations in time-continuous visuomotor tasks. This approach complements traditional trial-based paradigms by bridging controlled experimental research with real-world cognition. Our results demonstrate that cognitive neuroscience can benefit from advances in deep learning and encourage a broader methodological exchange between neuroscience and machine learning. This synergy is likely to become even stronger as machine learning models continue to grow in complexity and capability in the near future.

## Supplementary Material

Supplementary Material

## Data Availability

All behavioral data, the recorded screen observed by the subjects, as well as the results of our analysis on single-subject and group-analysis level are publicly available at https://osf.io/9cwq4/ (DOI: 10.17605/OSF.IO/9CWQ4). The experimental tasks, the DQN code used to generate the features, and all analysis scripts are accessible via the public GitHub repository at https://github.com/SHaberland15/Arcade_DQN_Research_fMRI. A permanent DOI for the repository was created via Zenodo: 10.5281/zenodo.17085737.
