# Supplementary material for: Encoding neural representations of time-continuous stimulus-response transformations in the human brain with advanced deep neural networks
Source: Imaging Neurosci (Camb). 2026 Mar 16;4:IMAG.a.1142. doi: 10.1162/IMAG.a.1142 (PMC12994000; doi:10.1162/IMAG.a.1142)
Supplement: Supplementary Material [file IMAG.a.1142_supp.pdf]

Supplementary materials

Figure S1 Prediction accuracy of the three encoding models across the three games. Pearson correlation between the actual and predicted fMRI voxel time series for the three encoding models using features from the baseline DQN (top), Ape-X (middle), and SEED (bottom). Neurons from all layers were used as predictors for each of the three games: Breakout (left), Space Invaders (middle), and Enduro (right). Only statistically significant voxels are shown (FWE-corrected, $p < 0.05$). While Figure 2 shows the mean prediction accuracy across all games, this figure shows the results separately for each game.

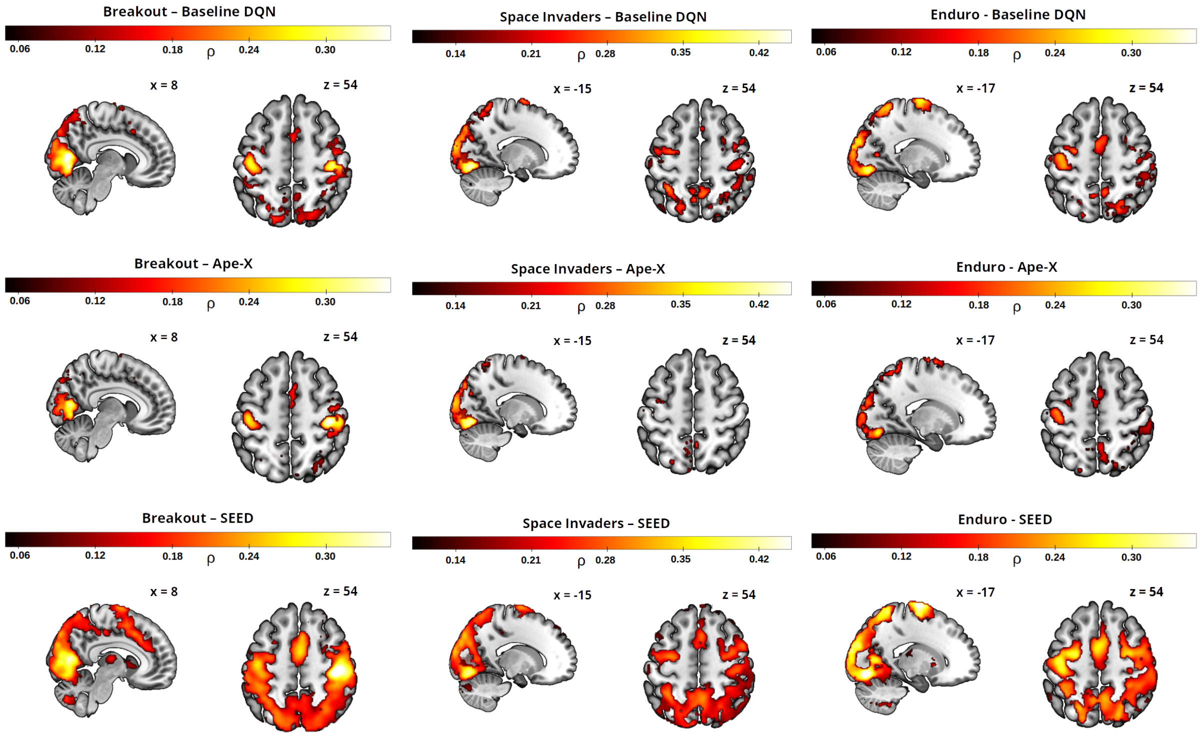

Figure S2 ROI-based analysis of voxels with significant prediction accuracy. The bar plots show the proportion of significantly predicted voxels (FWE-corrected, $p < 0.05$) within each ROI relative to the total number of voxels in that ROI. Encoding models were fitted using features from the baseline DQN (left), Ape-X (middle), and SEED (right), with neurons from the first, third, and fourth layers. The evaluated ROIs included V1/V2, PPC, M1, PMC, and LPFC.

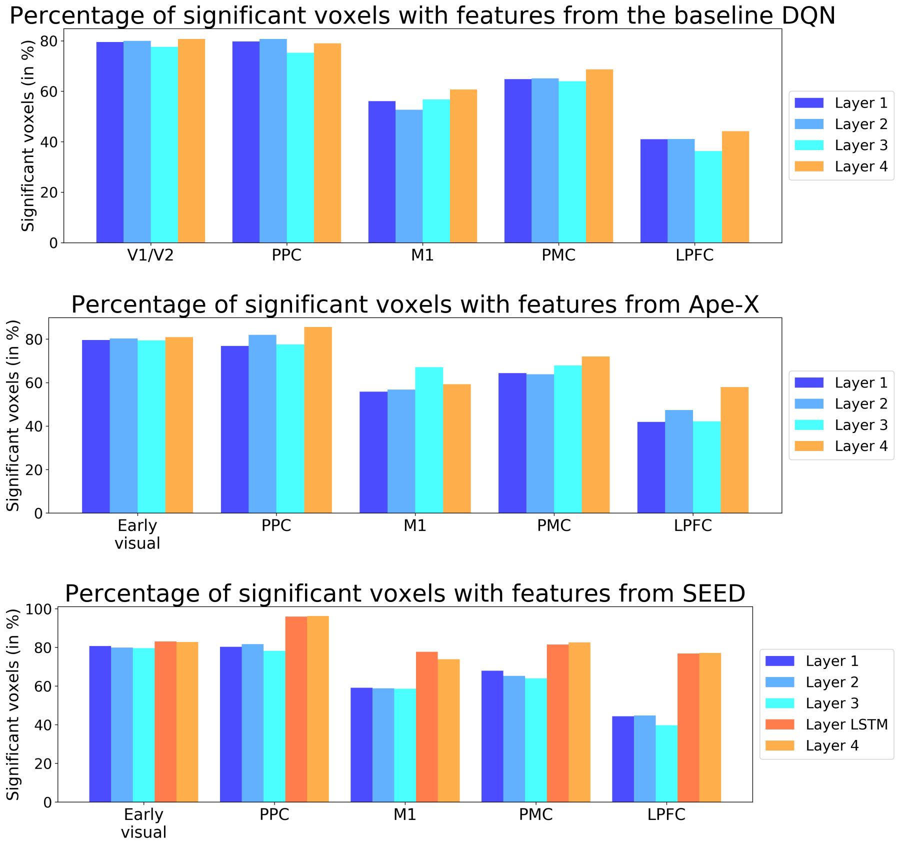

Figure S3 Hierarchical correspondence between the layers of a DQN and the stages of visuomotor processing. Pearson correlations were computed for voxels within V1/V2 and the PMC using features from the first and fourth layers of each DQN as predictors in the encoding model. Error bars represent the normalized 95% confidence interval of the mean correlation across subjects. The plotted lines are labeled with the gradient ρ(PMC) - ρ(V1/V2). A significant interaction between layer number and ROI was observed, reflected in an increase in the gradient with higher layer number. This increase is indicated by '**' (one-tailed paired t-test, p < 0.01). While prediction accuracy decreases from V1/V2 to PMC for all three DQNs, the decline was less pronounced in the fourth layer than in the first, with SEED showing the most prominent increse in the gradient (repeated-measures ANOVA, $F_{DQN}(1.75) = 76.01$, Greenhouse-Geisser corrected and post hoc test, p$<$ 0.01, Bonferroni-corrected). These findings, consistent with Section 3.3, provide preliminary evidence supporting a hierarchical correspondence between DQN layers and visuomotor processing stages in the human brain.

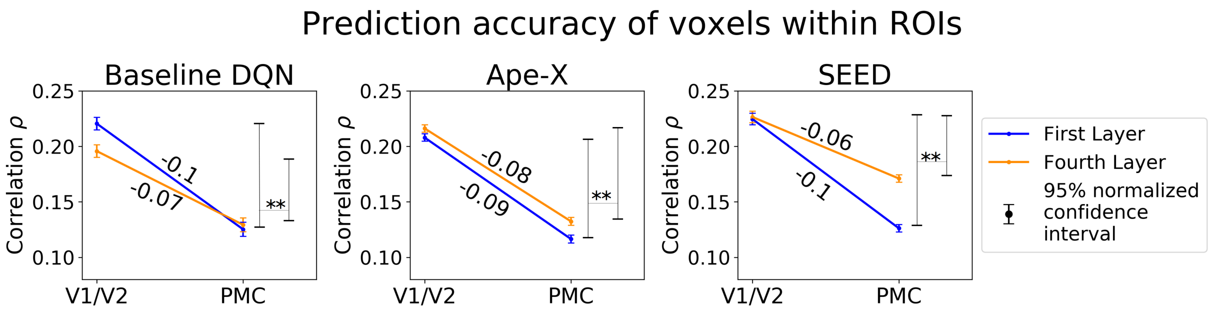

Figure S4 Contrasting prediction performance of early vs late layers of SEED. Voxel-wise prediction accuracies were compared between early (first, second, and third) layers and late (LSTM, fourth, and output) layers of SEED. The contrasts $[1, 1, 1, -1, -1, -1]$ (early $>$ late, shown in blue) and $[-1, -1, -1, 1, 1, 1]$ (late $>$ early, shown in orange) highlight voxels with significantly higher prediction accuracy in early and late layers, respectively. Color intensity represents the T-values of significant voxels (FWE-corrected, $p < 0.05$). Later layers provided more accurate predictions of voxel activity in higher-level PMC regions than earlier layers, whereas earlier layers captured voxel responses in V1/V2 more effectively than later layers.

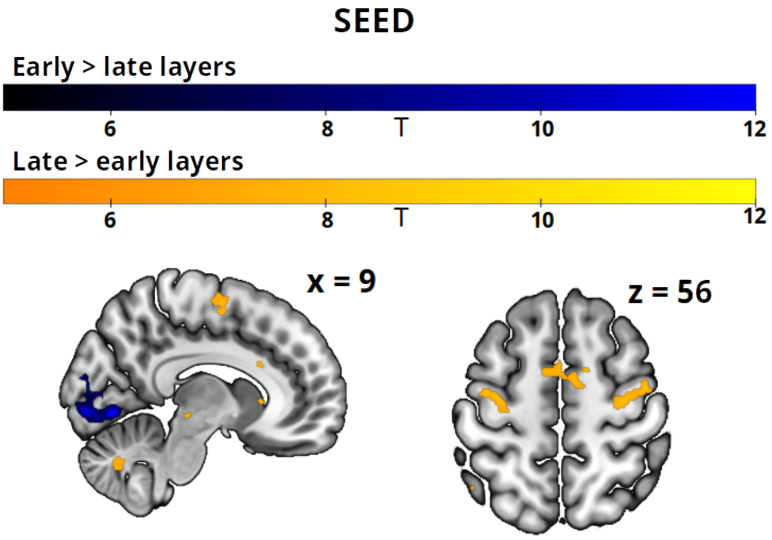

Figure S5 No hierarchical mapping between DQN layers and visual areas. The lines show the Pearson correlation for voxels in the visual areas V1, V2, V3, and V4. Features from the first (blue), second (green), and third (orange) convolutional layers of the DQNs were used as predictors in the encoding model. No clear hierarchical correspondence was found between the convolutional layers and the individual visual areas.

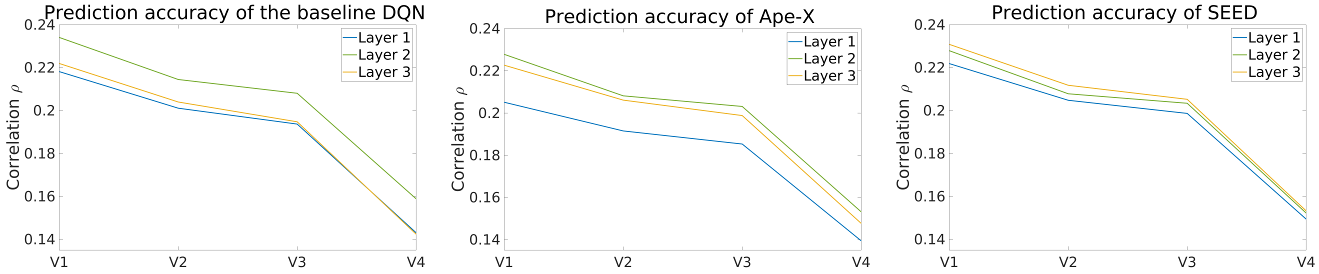

Figure S6 Relationship between behavioral and neural prediction accuracy. Each point represents a subject (N $= 22$), showing the average Pearson correlation for predicting human behavior in the form of motor responses and voxel-level neural activity along the dorsal stream. For each DQN, neurons from all layers were used as predictors. A positive relationship between behavior and neural activity was observed in all encoding models using features from the baseline DQN ($\rho = 0.49$), Ape-X ($\rho = 0.28$), and to a lesser extent, SEED ($\rho = 0.11$). These results suggest that while a model's output may align with behavioral data (see Supplementary Figure S11), this does not necessarily imply that the internal transformations from input to output closely mirror the underlying biological mechanisms.

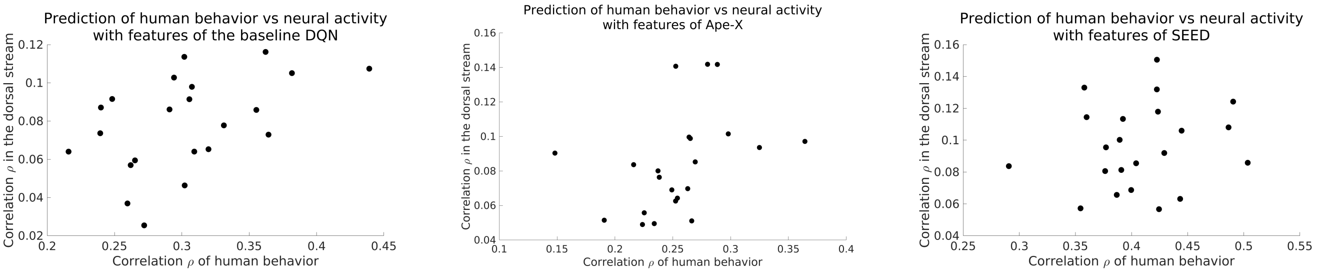

Figure S7 Differences in prediction accuracy by the fourth layer of the DQNs. Following the approach in Figure 4, this figure shows significant differences in Pearson correlation between actual and predicted neural activity (FWE-corrected, p < 0.05), computed using features from the fourth layer of SEED and those from the fourth layer of the baseline DQN (left), and those from SEED's fourth layer and the fourth layer of Ape-X (right). As shown in Section 3.2, significant differences in prediction accuracy among the DQN features became more pronounced in higher-level control regions. Features from SEED's fourth layer, the post-LSTM layer, more accurately predict activity in higher-level visual regions, including parts of the PPC and the PMC, compared to the fourth layers of the baseline DQN and Ape-X. These findings suggest that the LSTM may play a critical role. Its temporally integrated representations appear to enhance the model's ability to capture brain-related features, particularly in regions associated with higher cognitive control.

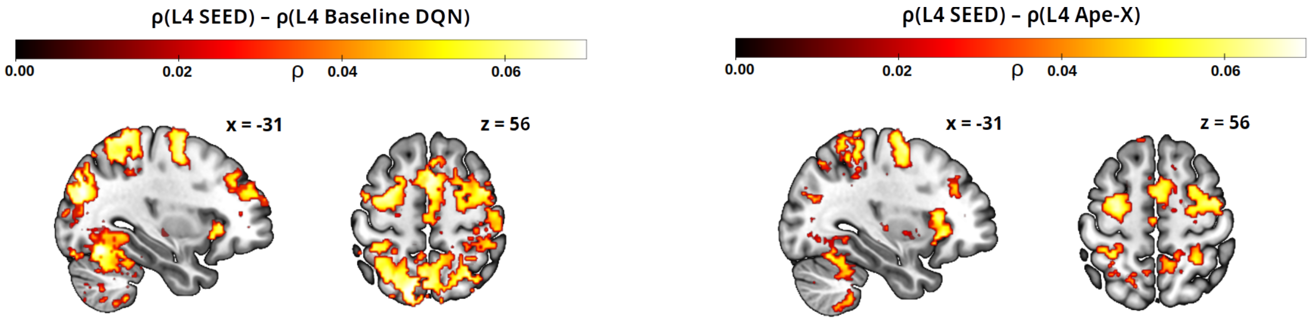

Figure S8 Differences in prediction accuracy by the first layer of the DQNs. Analogous to Supplementary Figure S7. Differences in Pearson correlation when predicting voxel activity using features from the first layer of SEED and those from the first layer of the baseline DQN (left), and from SEED's first layer and the first layer of Ape-X (right) (FWE-corrected, p < 0.05). This comparison highlights that the first convolutional layers yielded similar prediction performance, regardless of the subsequent network architecture.

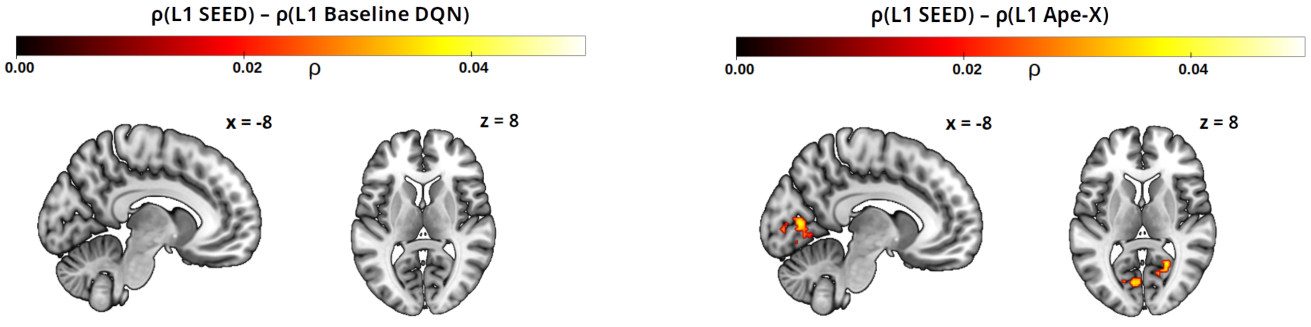

Figure S9 The role of training in developing effective feature generating mappings for neural prediction. For an untrained network, we used the architecture of the baseline DQN with randomly initialized weights, using three independent random initializations (light gray) on Breakout. We compared its predictive performance to that of the trained baseline DQN (dark gray), Ape-X (green), and SEED (red) in Breakout. Neurons from all layers were used as predictors in the GLM. Prediction performance was evaluated across V1/V2, PPC, M1, PMC, and LPFC. Error bars indicate the normalized 95% confidence interval of the mean Pearson correlation across subjects. As expected, the untrained DQN performed significantly worse (p < 0.001, Bonferroni-corrected), confirming that task-specific training is essential for extracting brain-relevant features and capturing functional representations during gameplay. Interestingly, even untrained networks with randomly initialized weights achieved above-chance prediction accuracy. This suggests that the architecture of a DQN itself, independent of its learned representations, contains inductive biases that align to some extent with features relevant for predicting neural responses. Similar findings have been reported in the same gameplay context, where DQN-generated features were used to model human behavior (Haberland et al., 2025), and in the domain of object recognition, where untrained DNNs were found to explain brain activity in visual areas (Cichy et al., 2016).

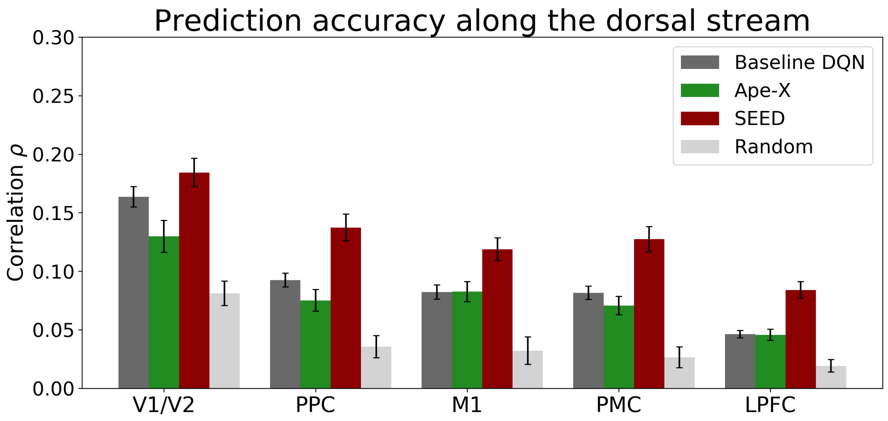

Figure S10 Challenges of using multiple DQN layers as predictors due to multicollinearity effects. We compared the prediction accuracy between layer-wise GLMs and GLMs using features from all layers. Bar plots show the Pearson correlation of the three encoding models in V1/V2, PPC, M1, PMC, and LPFC. Each ROI contains two bars: the left bar shows the encoding model using features from all layers of the corresponding DQN, while the right bar represents an encoding model using only features of a single specified DQN layer as predictors (the third layer of the baseline DQN and Ape-X, fourth layer of SEED). Error bars indicate the normalized 95% confidence interval of the mean Pearson correlation across subjects. Significant differences between the two GLMs were observed in all ROIs (two-sample t-test, p < 0.01, Bonferroni-corrected). The results highlight the impact of overfitting and multicollinearity, demonstrating that increasing the number of features in the GLM does not necessarily improve prediction performance. While this phenomenon may not hold for all layers, it is clearly observable for the ones examined in this analysis.

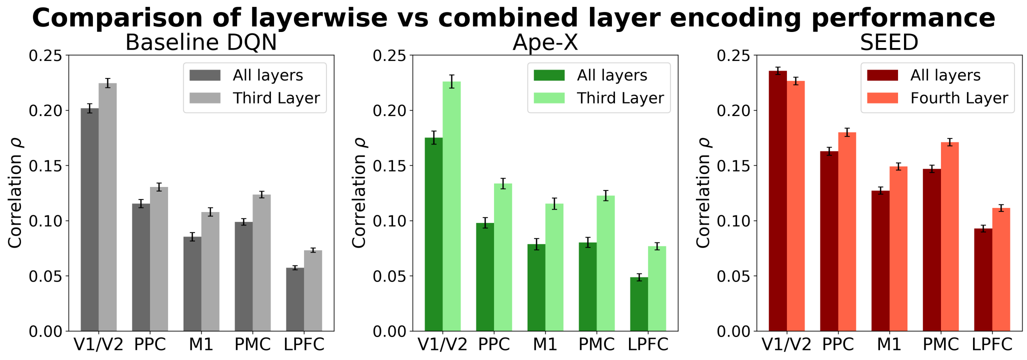

Figure S11 Prediction accuracy of human motor responses. Bars indicate the Pearson correlation between the actual motor responses of human subjects and the responses predicted with features of the baseline DQN (gray), Ape-X (green), and SEED (red), across Breakout (left), Space Invaders (middle), and Enduro (right). Since the output layer of a DQN contains Q-values for each possible action in a given state, these were used to predict behavior. Human behavior was predicted analogously to Haberland et al., 2025. A smoothing kernel of FWHM of 5.3 seconds was used, consistent with the smoothing used in the fMRI data. These results align with the original behavioral study of Haberland et al., 2025, showing that all three DQNs can generate features that predict human motor responses significantly above chance level (one-sample t-test, p < 0.001). Among them, SEED shows the highest prediction accuracy compared to the baseline DQN and Ape-X. Significant differences are marked with '***' (paired two-sample t-test, p < 0.001, Bonferroni-corrected) and '**' (paired two-sample t-test, p < 0.01, Bonferroni-corrected). Error bars indicate the normalized 95% confidence interval of the mean Pearson correlation across subjects. This analysis demonstrates that the encoding models can explain not only voxel activity but also behavioral data.

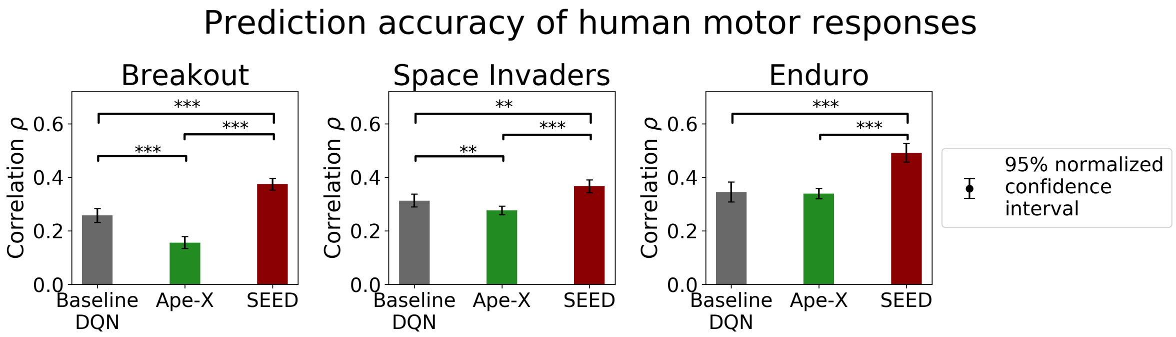

Figure S12 Differences in prediction accuracy between DQN-based encoding models and a control model using raw pixel values as predictors. The plots show significant differences in Pearson correlation between models using features derived from the DQNs and a control model based on the raw pixel values of the observed screens. Results are shown for the baseline DQN (left), Ape-X (middle), and SEED (right) (FWE-corrected, $p < 0.05$). Predictors included neural units from all layers of the DQNs.

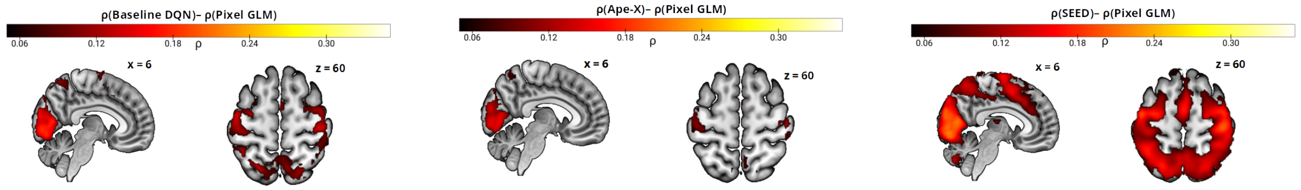

Figure S13 Differences in prediction accuracy between DQN-based encoding models and a control model using human motor responses as predictors. The plots show significant differences in Pearson correlation between models using features derived from the DQNs and the control model based on human motor responses. Results are shown for the baseline DQN (left), Ape-X (middle), and SEED (right) (FWE-corrected, $p < 0.05$). Neurons from all layers of the DQNs were used as predictors.

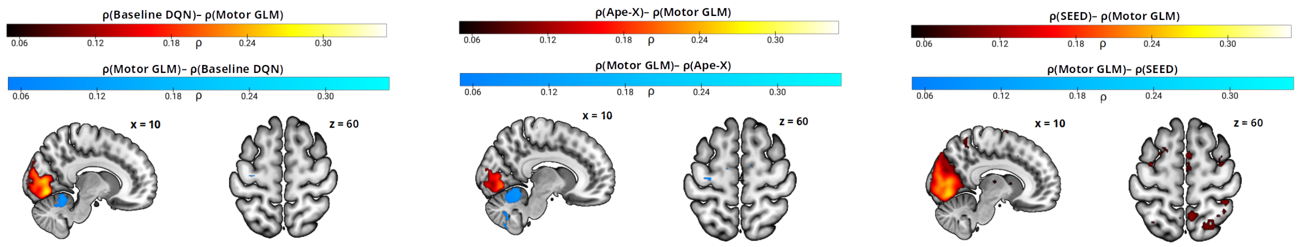

Figure S14 Learning curves of the DQNs. Adapted from Haberland et al. (2025). Shown are the learning curves for the baseline DQN (gray), Ape-X (green), and SEED (red). Since a pre-trained SEED model was used, only its performance is shown here; the full training curves are available on Espeholt et al. (2021).

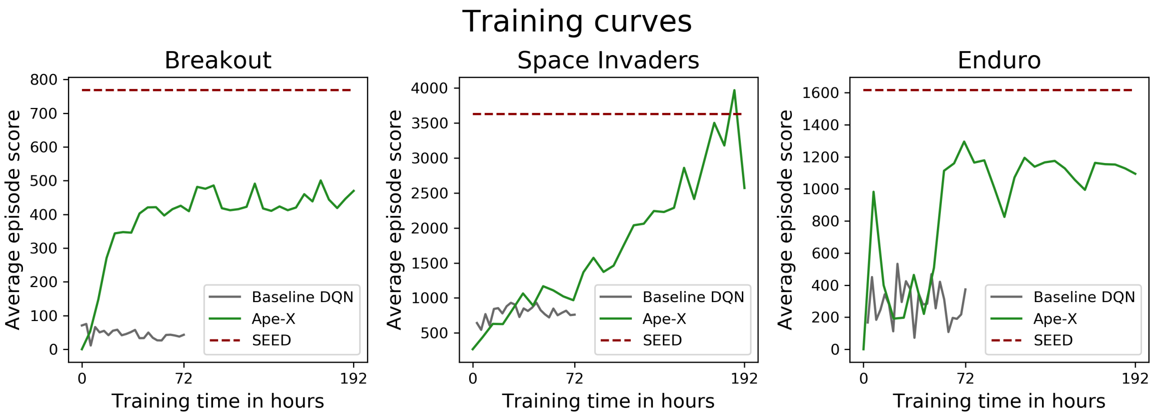

Figure S15 Visualization of the experimental gameplay. Participants played three Atari games Breakout (a), Space Invaders (b), and Enduro (c) using a four-button box (d; image taken from https://www.curdes.com). The screen arrays (original size: 160×210) were downsampled to an array of 84×84, ensuring that participants received the same input as the DQNs.

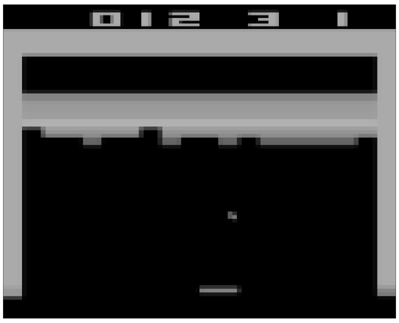

(a)

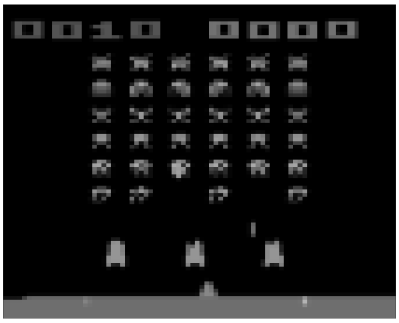

(b)

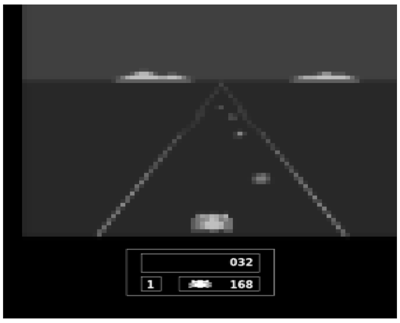

(c)

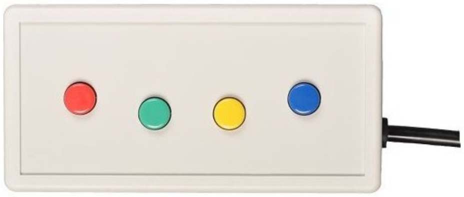

(d)

Figure S16 Gaming performance of subjects vs. neural prediction performance. Scatter plots illustrating the relationship between subjects' gaming performance and the DQN-based prediction accuracy of neural activity in the dorsal stream. Each point represents an individual subject. 785 786 787

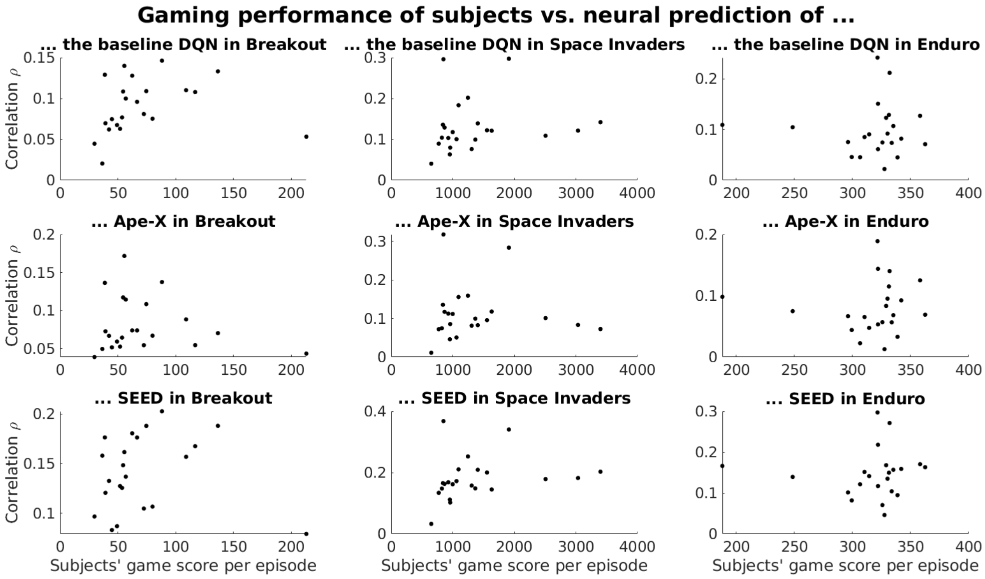

Figure S17 Prediction accuracy in the ventral vs dorsal stream. Bar plots show the voxel-wise Pearson correlations in ventral (MTG, IT) and dorsal (precuneus, SPL, M1, and PMC) regions, averaged across all voxels within each ROI. The encoding models used features from all layers of the baseline DQN (left), Ape-X (middle), and SEED (right). Error bars represent the normalized 95% confidence interval of the mean correlation across subjects. Prediction accuracies were significantly higher in the dorsal visual stream compared to the ventral visual stream (two-sample t-test, p < 0.001; significance denoted by '***'). We note, however, that this comparison should be interpreted with caution, as differences in ROI characteristics, such as size and signal quality, could influence the observed effect.

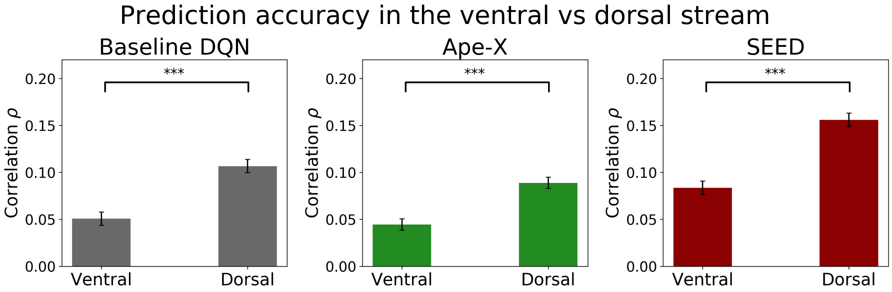

Table S1 Effect of L1-regularization on multicollinearity among predictors. To examine the effect of L1-regularization, we assessed several metrics quantifying multicollinearity. We investigated the design matrix A, used to predict the fMRI time series of voxel activity, consisted of columns derived from the first layer of SEED, generated during gameplay of Breakout. To evaluate multicollinearity, we computed the number of regressors in A, the rank of A, the determinant $det(A^T A)$, the condition number $\kappa(A^T A)$, and the maximum value in the column-wise correlation matrix of A. We compared three different L1-regularization strengths to the case without regularization. Since GLMs were estimated voxel-wise, we randomly selected 1000 significant voxels (FWE-corrected, p < 0.05) and averaged the measures of multicollinearity across these voxels, as well as across the five sessions and participants. The results clearly demonstrate that increasing regularization reduces multicollinearity, as expected, highlighting the important role of regularization. In the main analyses, we used a regularization parameter of $\lambda = 0.07$ for layer-wise prediction performance, and $\lambda = 0.2$ for the prediction of DQNs where features from all layers were combined as predictors in the encoding model.

	$\lambda = 0$	$\lambda = 0.005$	$\lambda = 0.01$	$\lambda = 0.07$
**Number regressors**	11371.57	183.52	133.12	46.34
**Rank**	415.65	183.48	133.10	46.34
**Determinant**	0	4.46e+271	9.47e+259	1.05e+151
**Condition number**	4.99e+50	7.22e+15	1.09e+17	2.28e+12
**Correlation**	1	0.98	0.97	0.85

Table S2 Effect of L1-regularization on multicollinearity among predictors. Same as in Supplementary Table S1 with SEED's fourth layer.

	$\lambda = 0$	$\lambda = 0.005$	$\lambda = 0.01$	$\lambda = 0.07$
**Number regressors**	676.22	93.46	70.24	18.63
**Rank**	415.65	93.46	70.24	18.63
**Determinant**	0	7.04e+185	2.17e+164	9.38e+78
**Condition**	1.09e+128	1.69e+13	1.33e+13	1863.88
**Correlation**	1	0.94	0.90	0.74

Table S3 Multicollinearity between the predictors from two feature sets. To assess the relationship between predictors from two different layers, we used Canonical Correlation Analysis (CCA) to quantify the dependency between feature sets derived from the first and fourth layers of SEED during gameplay in Breakout. CCA identifies linear combinations of variables within each feature set such that the correlation between the resulting combinations is maximized, capturing the shared variance between the two sets. The table displays the top five canonical correlations for each tested regularization parameter. The analysis was conducted on 1000 randomly selected significant voxels (FWE-corrected, $p < 0.05$) and averaged across voxels, sessions, and participants. This analysis demonstrates that regularization effectively reduces the dependency between feature representations from different layers. It also emphasizes the inherent correlation between layers resulting from the feedforward architecture, where later layers build directly upon the representations of earlier ones. These interdependencies are particularly relevant for the GLM analyses, in which features from all layers were used simultaneously as predictors. In such cases, multicollinearity among predictors can lead to reduced encoding model performance compared to GLMs based on features from individual layers (see Supplementary Figure S10).

$\lambda = 0.005$	$\lambda = 0.01$	$\lambda = 0.07$
1	1	0.97
1	1	0.95
1	1	0.93
1	1	0.92
1	1	0.84

Table S4 Custom contrast analysis for the interaction effect ROI × DQN in Figure 3.

ROI	DQN	PPC > V1/V2	PMC > V1/V2	PPC > M1	PMC > M1
V1/V2	Baseline DQN	1	1	0	0
PPC		−1	0	−1	0
M1		0	0	1	1
PMC		0	−1	0	−1
LPFC		0	0	0	0
V1/V2	Ape-X	1	1	0	0
PPC		−1	0	−1	0
M1		0	0	1	1
PMC		0	−1	0	−1
LPFC		0	0	0	0
V1/V2	SEED	−2	−2	0	0
PPC		2	0	2	0
M1		0	0	−2	−2
PMC		0	2	0	2
LPFC		0	0	0	0

Table S5 Results of the custom contrast for ROI × DQN.

Contrast	Estimate	SE	df	t	p	Cohen's d
PPC > V1/V2	0.015	0.006	21.000	2.461	0.023	0.363
PMC > V1/V2	0.017	0.006	21.000	2.899	0.009	0.406
PPC > M1	0.023	0.006	21.000	3.727	0.001	0.555
PMC > M1	0.025	0.004	21.000	5.749	< 0.001	0.598

Table S6 Custom contrast analysis for the interaction effect DQN $\times$ Layer in Figure 4.

DQN	Layer	Differences in layer 4 $>$ layer 1 and 3
Baseline DQN	Layer 1	0.500
Ape-X		0.500
SEED		−1.000
Baseline DQN	Layer 3	0.500
Ape-X		0.500
SEED		−1.000
Baseline	Layer 4	−1.000
Ape-X		−1.000
SEED		2.000
Baseline DQN	Output	0.000
Ape-X		0.000
SEED		0.000

Table S7 Results of the custom contrast for DQN $\times$ Layer.

Contrast	Estimate	SE	df	t	p	Cohen's d
1	0.055	0.004	21.000	14.304	< 0.001	1.502

Table S8 Custom contrast analysis for the interaction effect DQN × ROI for the fourth layer in Figure 4.

DQN	ROI	PMC > M1	PMC > V1/V2	PPC > M1	PPC > V1/V2
Baseline DQN	V1/V2	0	1	0	1
Ape-X		0	1	0	1
SEED		0	−2	0	−2
Baseline DQN	PPC	0	0	−1	−1
Ape-X		0	0	−1	−1
SEED		0	0	2	2
Baseline DQN	M1	1	0	1	0
Ape-X		1	0	1	0
SEED		−2	0	−2	0
Baseline DQN	PMC	−1	−1	0	0
Ape-X		−1	−1	0	0
SEED		2	2	0	0
Baseline DQN	LPFC	0	0	0	0
Ape-X		0	0	0	0
SEED		0	0	0	0

Table S9 Results of the custom contrast for DQN × ROI for the fourth layer.

Contrast	Etimate	SE	df	t	p	Cohen's d
PMC > M1	0.018	0.004	21.000	4.168	< 0.001	0.469
PMC > V1/V2	0.039	0.005	21.000	8.615	< 0.001	1.018
PPC > M1	0.025	0.006	21.000	4.101	< 0.001	0.645
PPC > V1/V2	0.046	0.005	21.000	8.500	< 0.001	1.194

Table S10 Statistical results for the comparison of early and late layer assignments in Figure 5. Post hoc comparisons for the factor ROI. Layer assignment analysis from Section 3.3 using the log-ratio transformation described in Greenacre, 2021 to account for compositional data. Statistical test on the ratio of early layers (layers 1-3) to late layers (LSTM, layer 4) across the ROIs.

		Mean Difference	SE	df	t	p_{bonf}	Cohen's d
V1/V2	PPC	1.885	0.171	21	11.017	< 0.001	2.025
	M1	1.369	0.157	21	8.724	< 0.001	1.470
	PMC	2.449	0.172	21	14.240	< 0.001	2.630
	LPFC	1.702	0.150	21	11.330	< 0.001	1.828
PPC	M1	−0.516	0.229	21	−2.257	0.348	−0.554
	PMC	0.564	0.186	21	3.029	0.064	0.605
	LPFC	−0.183	0.185	21	−0.993	1.000	−0.197
M1	PMC	1.080	0.154	21	6.999	< 0.001	1.160
	LPFC	0.333	0.158	21	2.112	0.468	0.357
PMC	LPFC	−0.747	0.124	21	−6.006	< 0.001	−0.802

Table S11 Significant brain regions predicted by the DQNs, corresponding to Figure 2. Overview of significant cluster-level results from the SPM analysis for predicting neural activity by the encoding model using features from the baseline DQN. Reported are the corresponding brain regions (anatomical labels according to the AAL2 atlas), peak MNI coordinates (x, y, z), cluster size (k), and peak-level t-values. The p-values were FWE-corrected at the cluster-level ($p < 0.0001$) with an extent threshold of 30 voxels. Compared to the main analyses, the FWE threshold was set more conservatively here for improved clarity.

Region	x	y	z	k	t-value (peak)
Lingual_R	10	-72	-6	4493	27.40
Postcentral_R	44	-38	60	779	17.12
Parietal_Sup_R	16	-64	62	516	16.90
Precentral_L	-40	-20	54	386	16.65
Precentral_L	-32	-6	60	169	13.50
Insula_R	44	16	-2	31	13.47
SupraMarginal_R	58	-42	42	47	13.13
Temporal_Mid_L	-50	-70	0	103	13.07
SupraMarginal_R	58	-30	40	62	12.96
Supp_Motor_Area_R	6	0	74	40	12.86

Table S12 Significant brain regions predicted by the DQNs, corresponding to Figure 2. Overview of significant cluster-level results from the SPM analysis for predicting neural activity by the encoding model using features from Ape-X. Reported are the corresponding brain regions (anatomical labels according to the AAL2 atlas), peak MNI coordinates (x, y, z), cluster size (k), and peak-level t-values. The p-values were FWE-corrected at the cluster-level ($p < 0.0001$) with an extent threshold of 30 voxels. Compared to the main analyses, the FWE threshold was set more conservatively here for improved clarity.

Region	x	y	z	k	t-value (peak)
Calcarine_L	-8	-84	2	2938	28.55
Postcentral_L	-40	-22	54	183	16.19
Postcentral_R	50	-24	54	262	14.63
Frontal_Sup_2_R	26	-4	66	30	13.74
Occipital_Mid_L	-46	-68	0	71	12.83
Precuneus_R	4	-54	64	54	12.57

Table S13 Significant brain regions predicted by the DQNs, corresponding to Figure 2. Overview of significant cluster-level results from the SPM analysis for predicting neural activity by the encoding model using features from SEED. Reported are the corresponding brain regions (anatomical labels according to the AAL2 atlas), peak MNI coordinates (x, y, z), cluster size (k), and peak-level t-values. The p-values were FWE-corrected at the cluster-level ($p < 0.0001$) with an extent threshold of 50 voxels. Compared to the main analyses, the FWE threshold was set more conservatively here for improved clarity.

Region	x	y	z	k	t-value (peak)
Lingual_R	10	-72	-8	24366	31.67
Insula_R	34	20	6	485	17.69
Angular_R	56	-60	34	65	16.40
Precentral_R	58	8	36	241	16.29
Angular_R	-52	-66	36	136	16.24
Cingulate_Mid_L	-10	-26	40	78	15.93
Frontal_Sup_Medial_L	-10	34	58	71	15.89
Thalamus_R	10	-12	10	143	14.37
Frontal_Inf_Orb_2_L	-48	40	-8	59	14.26
Cerebellum_9_L	-18	-42	-48	51	14.12
Frontal_Sup_2_R	16	50	38	180	14.07
Putamen_L	-24	8	4	142	13.32
Cerebellum_9_L	-8	-56	-56	96	13.26
Frontal_Inf_Tri_R	46	38	2	53	13.03

Table S14 Significant brain regions with differences between encoding models, corresponding to Figure 3. Overview of significant cluster-level results from the SPM analysis for differences in prediction accuracy between the encoding models using features from the baseline DQN and Ape-X. Reported are the corresponding brain regions (anatomical labels according to the AAL2 atlas), peak MNI coordinates (x, y, z), cluster size (k), and peak-level t-values. The p-values were FWE-corrected at the cluster-level ($p < 0.05$) with an extent threshold of 5 voxels.

Region	x	y	z	k	t-value (peak)
Frontal_Sup_2_L	-24	-8	64	7	8.41
Occipital_Mid_L	-24	-84	26	7	8.12

Table S15 Significant brain regions with differences between encoding models, corresponding to Figure 3. Overview of significant cluster-level results from the SPM analysis for differences in prediction accuracy between the encoding models using features from SEED and the baseline DQN. Reported are the corresponding brain regions (anatomical labels according to the AAL2 atlas), peak MNI coordinates (x, y, z), cluster size (k), and peak-level t-values. The p-values were FWE-corrected at the cluster-level ($p < 0.05$) with an extent threshold of 50 voxels.

Region	x	y	z	k	t-value (peak)
Supp_Motor_Area_R	4	8	56	22230	21.68
Frontal_Inf_Tri_L	-36	20	8	439	13.96
Insula_R	34	26	-4	1422	13.61
Caudate_L	-16	0	20	652	13.35
Frontal_Sup_2_R	24	56	28	192	13.09
Postcentral_L	-62	-12	30	394	12.26
Cerebellum_8_R	36	-48	52	55	11.64
Temporal_Mid_R	62	-38	-8	50	10.62
Cerebellum_8_R	16	-66	-46	83	10.26
Frontal_Sup_2_L	-26	34	28	63	10.06
Parietal_Inf_L	-44	-50	38	73	9.84

Table S16 Significant brain regions with differences between encoding models, corresponding to Figure 3. Overview of significant cluster-level results from the SPM analysis for differences in prediction accuracy between the encoding models using features from SEED and Ape-X. Reported are the corresponding brain regions (anatomical labels according to the AAL2 atlas), peak MNI coordinates (x, y, z), cluster size (k), and peak-level t-values. The p-values were FWE-corrected at the cluster-level ($p < 0.05$) with an extent threshold of 50 voxels.

Region	x	y	z	k	t-value (peak)
Calcarine_L	-12	-66	12	33828	19.27
Frontal_Inf_Tri_L	-40	18	6	1557	15.06
Frontal_Mid_2_L	-38	36	30	276	11.73
Frontal_Mid_2_R	34	36	44	471	11.42
Putamen_L	-28	-20	4	60	11.03
Lingual_R	6	-40	2	60	9.85
